# Hydrographic and sea ice gradients shape benthic ecosystem functioning through sediment characteristics and benthic functional structure in Nares Strait, Canadian Arctic

**DOI:** 10.1371/journal.pone.0345559

**Published:** 2026-11-13

**Authors:** Thibaud Combaz, Bodil A. Bluhm, Ursula Witte, Philippe Archambault

**Affiliations:** 1 Takuvik International Research Laboratory - CNRS - Sorbonne Université, Université Laval, Québec, QC, Canada; 2 Department of Arctic and Marine Biology, UiT The Arctic University of Norway, Tromsø, Norway; 3 Institute for Biological and Ecological Sciences, University of Aberdeen, Aberdeen, United Kingdom; Universidade de Aveiro, PORTUGAL

## Abstract

Rapid changes in Arctic sea ice conditions are altering the quantity, quality, and timing of organic matter reaching the seafloor, thereby reshaping benthic ecosystem functioning. However, the exact mechanisms linking sea ice variability, benthic community structure, and sediment biogeochemical processes remain poorly understood. With its multiple forms and ages of sea ice, Nares Strait in the Canadian Arctic offers a unique natural laboratory for investigating how environmental gradients and benthic community structure jointly regulate benthic ecosystem functioning. Here, we incubated sediment cores from several locations in Nares Strait characterised by different sea ice conditions, ranging from first-year ice to multi-year ice, and measured oxygen and nutrient fluxes as proxies for ecosystem functioning. To investigate the relative importance of potential drivers, we also measured environmental variables, identified macrofauna and calculated a suite of taxonomic and functional diversity indices. Our analyses show that fluxes across the sediment-water interface varied significantly between the northern and southern regions of Nares Strait. Among individual predictors, the average proportion of deposit feeders and sea ice cover (days since ice-free) explained the largest proportions of flux variability (23% and 14%, respectively). Overall, benthic functional structure explained more variation in fluxes than taxonomic diversity. Environmental variables explained little additional variation once functional descriptors were included, while variance partitioning revealed that the shared environmental-functional component accounted for 25% of the total variation, representing more than half of the variation explained by out models (45%) and exceeding the unique contributions of either environmental or functional variables. This strong overlap indicates that benthic functional structure integrates the cumulative effects of environmental forcing on sediment biogeochemical functioning. Collectively, our findings suggest that hydrographic and sea ice gradients regulate benthic ecosystem functioning largely through their influence on sediment characteristics and benthic functional structure. Because Nares Strait encompasses environmental conditions representative of many Arctic marine systems, the mechanisms identified here provide a framework for predicting how continued sea ice decline may reshape benthic ecosystem functioning across the Arctic Ocean.

## Introduction

Marine soft-sediment ecosystems play a fundamental role in benthic ecosystem functioning globally by regulating the cycling and long-term storage of carbon and nutrients. Following the deposition of organic matter primarily from the water column, sediments become sites of intense microbial and faunal activity where organic matter is degraded through a succession of redox reactions, while a small remaining part is permanently buried [1]. Organic matter degradation proceeds through a succession of microbial metabolic pathways that use progressively less energetically favorable terminal electron acceptors, including oxygen, nitrate, manganese and iron oxides, and sulfate [2]. The relative importance of these pathways is governed by the balance between oxygen supply and organic matter mineralization demand, which establishes sediment redox conditions and ultimately regulates carbon and nutrient cycling.

The relative contributions of these different pathways are controlled by a complex interplay of environmental and biological factors. Environmental variables such as organic matter supply, temperature, salinity and sediment characteristics regulate oxygen penetration depth and sediment redox conditions, thereby regulating the relative importance of the microbial metabolic pathways responsible for carbon and nutrient cycling [3–5]. At the same time, benthic organisms modify sediment biogeochemistry through feeding activities, sediment reworking, and bioirrigation, directly influencing oxygen transport, redox conditions, microbial processes, and nutrient regeneration [6,7]. Consequently, oxygen and nutrient exchanges across the sediment–water interface integrate the combined effects of environmental forcing, sediment biogeochemistry, and benthic community activity, making them integrative indicators of benthic ecosystem functioning.

These interactions are particularly relevant in the Arctic Ocean, where strong seasonal variability in sea ice cover governs primary production, pelagic-benthic coupling, and the delivery of organic matter to the seafloor [8–11]. Rapid Arctic warming is profoundly modifying these processes. Arctic-wide open-water area has already increased by approximately 27% since the late 1990s, while continued reductions in sea ice extent and thickness are expected throughout the twenty-first century [11–13]. These changes are likely to alter sediment characteristics, oxygen availability, nutrient cycling, and carbon storage, thereby reshaping benthic ecosystem functioning and the capacity of Arctic sediments to process and store organic carbon [14,15]. However, despite the recognized importance of environmental forcing, the relative importance of environmental conditions and benthic community structure in controlling sediment biogeochemical functioning remains poorly understood.

Benthic organisms represent a key link between environmental conditions and sediment biogeochemistry. Through feeding activities, sediment reworking and enhanced porewater exchange, they modify oxygen availability and nutrient cycling while responding to long-term changes in habitat quality and food supply. Increasing evidence suggests that functional diversity provides a more mechanistic understanding of these processes than taxonomic diversity alone because it reflects the ecological roles performed by organisms [16–19]. Because functional traits of benthic fauna determine how organisms interact with sediments and process organic matter, they are expected to provide a closer mechanistic link to ecosystem functioning than taxonomic identity alone. Traits related to feeding strategy, mobility, body size and sediment reworking are particularly relevant because they influence organic matter processing, sediment mixing, bioirrigation, and the exchange of oxygen and nutrients across the sediment–water interface [20]. However, relatively few studies have simultaneously evaluated the respective contributions of environmental conditions, taxonomic diversity, and functional diversity to benthic ecosystem functioning under contrasting Arctic sea ice regimes.

Nares Strait, located between western Greenland and Canada’s eastern part of the Arctic, provides an ideal natural laboratory to investigate these contributions. The strait forms a major connection between the Central Arctic Ocean and northern Baffin Bay and encompasses a broad spectrum of Arctic sea ice and hydrographic regimes. It extends from the Lincoln Sea in the north, where some of the oldest and thickest remaining sea ice in the Arctic Ocean is found, to the North Water Polynya (*Pikialasorsuaq*; NOW; Fig 1) in the south, one of the most productive marine regions in the Arctic [21–23]. Nares Strait is also part of the Last Ice Area, a region expected to retain summer sea ice longer than most of the Arctic Ocean as climate warming progresses [24]. The pronounced gradients in sea ice conditions, primary production, currents and sedimentary environments make Nares Strait a unique system for investigating how environmental variability influences benthic ecosystem functioning. Moreover, because the region encompasses environmental conditions representative of many Arctic marine systems, it provides valuable insights into how ongoing sea ice decline may affect benthic ecosystem functioning throughout the Arctic Ocean.

**Fig 1:**
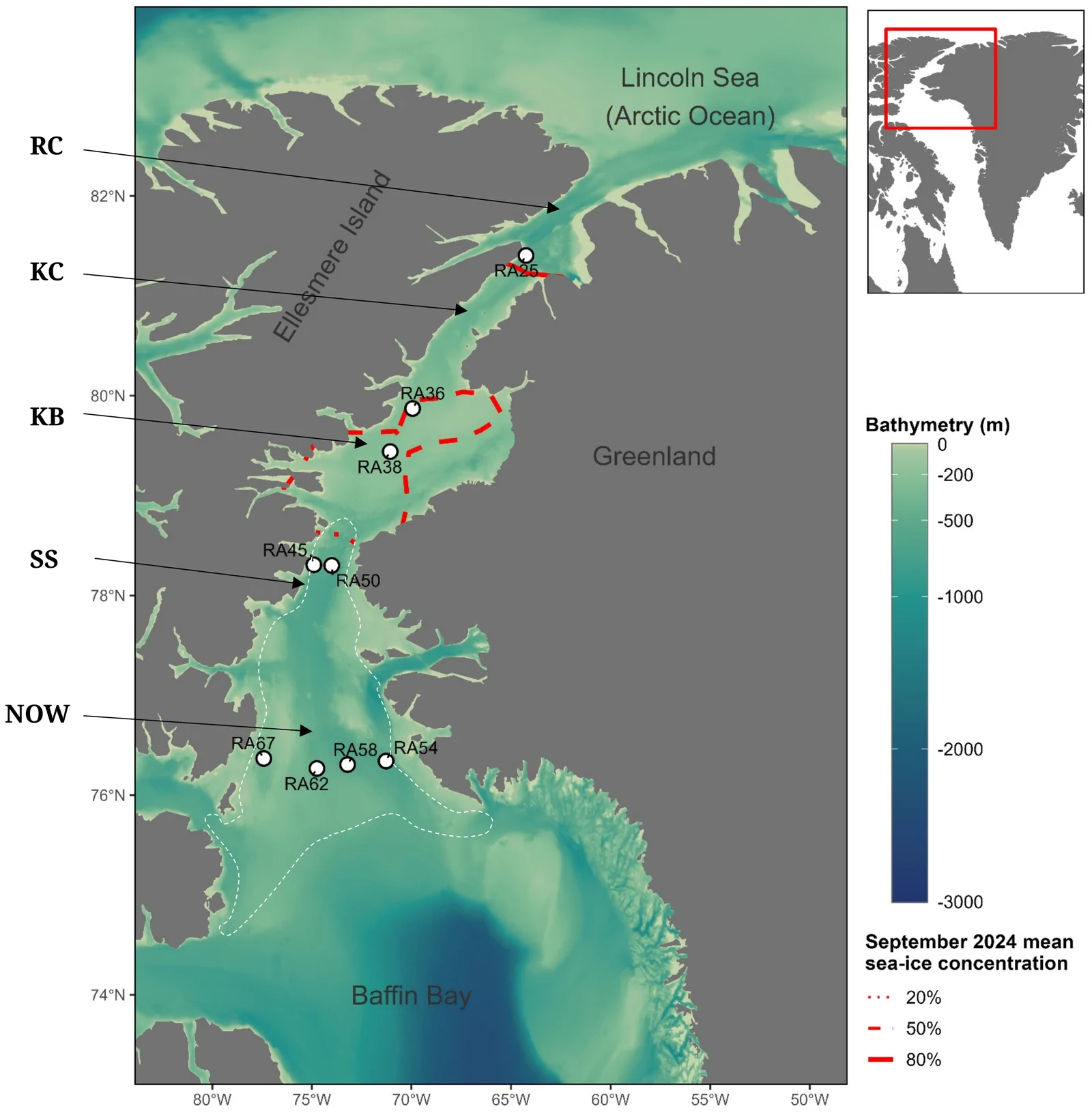
Location of stations sampled for sediment incubations in Nares Strait between Arctic Canada and Greenland in September 2024. Study area in Nares Strait showing sampling stations in September 2024, bathymetry, and mean September 2024 sea-ice concentration contours (20%, 50% and 80%). Bathymetry is from the GEBCO_2026 Grid; coastline from Natural Earth; sea-ice concentration contours derived from the NOAA/NSIDC Climate Data Record; the North Water Polynya extent is based on Inuit Circumpolar Council Pikialasorsuaq Commission (2017). Abbreviations represent locations along the strait: Robeson Channel (RC); Kennedy Channel (KC); Kane Basin (KB); Smith Sound (SS) and North Water Polynya (NOW).

The objectives of this study were therefore to (i) quantify oxygen and nutrient fluxes across the sediment-water interface in the different subregions of Nares Strait, (ii) identify the potential environmental and diversity drivers of benthic ecosystem functioning, and (iii) determine the relative contributions of environmental conditions, taxonomic diversity, and functional diversity to sediment-water interface flux variability.

## Materials and methods

### Study site

Nares Strait is a narrow passage along which sea ice and freshwater leave the Arctic Ocean southward into sub-Arctic waters and is the second most important outflow for sea ice next to the outflow in western Fram Strait. The strait is bordered by Greenland to the east and by Ellesmere Island to the west. It is essentially a flow-through system, consisting of a series of deep channels and shallower basins (from north to south: Robeson Channel, Kennedy Channel, Kane Basin and Smith Sound; Fig 1).

From September to June, more than 80% of Nares Strait is covered by sea ice [25]. The magnitude and the timing of sea ice export through the strait largely depends on ice arches that usually form at the northern and southern end of the strait and can remain stable for months at a time, preventing mobile ice from flowing southward [26,27]. These ice arches also determine the timing, location, and intensity of phytoplankton blooms by modulating ice export, stratification and light penetration [27]. Depending on the configuration of the ice arches, two productivity hotspots were identified in the region: the first in Kennedy Channel and the second in the NOW in northern Baffin Bay (Fig 1) [27]. Nares Strait is also affected by strong southward currents originating from the Arctic Ocean and driven by persistent northerly winds channeled through the steep topography of the strait. Together with water depth and proximity to the coast, these currents strongly influence sediment transport and sorting, resulting in marked spatial variability in sediment characteristics [28]. Sediments generally range from medium silt to fine sand (24–136 µm), which is relatively coarse compared with sediments in many other Canadian Arctic shelf areas. Sediments in Nares Strait are characterized by among the lowest total organic carbon (TOC) contents reported for the Canadian Arctic [28].

### Field sampling

Nine stations were sampled in September 2024 during the RefugeArctic cruise aboard the research icebreaker CCGS Amundsen along a latitudinal gradient in Nares Strait in the Canadian Arctic (Fig 1 and Table 1). Research licenses were obtained from the Department of Fisheries and Ocean Sciences to conduct research in the Canadian part of Nares Strait (Permit #S-24/25-1040-NU) and from the Danish Ministry of Foreign Affairs for the Greenlandic part (Permit #2024/034776). The three northernmost stations, characterized by permanent landfast ice, were located in Kennedy Channel (RA25) and Kane Basin (RA36 and RA38). Two other stations with seasonal landfast sea ice coverage were located in Smith Sound (RA45 and RA50). The remaining four southernmost stations were located in northern Baffin Bay and permanently free of ice (RA54, RA58, RA62 and RA67).

**Table 1:** Environmental and biological characteristics of stations sampled for biogeochemical incubations in Nares Strait in 2024.

Region	Station	Date sampled (mm-dd-2024)	Latitude (°N)	Water depth (m)	Bottom water temperature (°C)	Salinity (psu)	Days since ice-free	Bottom DO (µM)	Mean grain size (μm)	OM content (%)	Chl *a* (μg g^−1^ DM)	Chl*a*:phaeo ratio	Abundance (ind. core^-1^)	Biomass (g WM core^-1^)	Taxa richness (taxon core^-1^)	Functional richness
**Kennedy Chanel**	RA25	09-08	81.404	516.4	0.3	34.7	0	276.6	26.5	1.4	0.1 ± 0.1	0.1	4.3 ± 1.5	0.01 ± 0.01	2 ± 1	1.3 ± 0.0
**Kane Basin**	RA36	09-13	79.872	221.0	0.0	34.5	39	271.3	35.6	7.8	0.1 ± 0.0	0.2	6.0 ± 0.0	0.08 ± 0.06	4.3 ± 0.6	9.4 ± 2.9
	RA38	09-15	79.441	223.1	0.0	34.5	0	270.3	29.7	2.8	1.5 ± 1.2	0.2	32.0 ± 7.5	0.12 ± 0.04	7.3 ± 1.2	17.0 ± 2.2
**Smith Sound**	RA45	09-18	78.307	550.2	-0.2	34.2	90	269.2	27.0	2.0	0.4 ± 0.1	0.1	18.7 ± 8.6	0.19 ± 0.09	11.3 ± 2.5	16.6 ± 3.0
	RA50	09-20	78.300	601.7	0.2	34.2	92	258.0	29.4	3.4	0.8 ± 0.4	0.1	45.7 ± 18.1	0.18 ± 0.09	11.3 ± 3.8	11.0 ± 2.6
**Northern Baffin Bay**	RA54	09-23	76.342	657.0	1.5	34.5	46	202.5	17.9	6.8	1.1 ± 0.5	0.1	329.3 ± 22.5	0.35 ± 0.01	10.0 ± 1.0	13.0 ± 3.7
	RA58	09-24	76.307	591.3	0.3	34.3	40	247.5	17.7	5.3	2.9 ± 1.9	0.1	44.3 ± 10.7	0.15 ± 0.02	8.7 ± 2.1	16.3 ± 1.3
	RA62	09-25	76.267	457.5	0.3	34.3	83	246.7	23.5	5.7	1.1 ± 0.3	0.2	201.7 ± 21.0	0.29 ± 0.01	6.3 ± 1.2	7.2 ± 2.1
	RA67	09-26	76.368	388.6	0.2	34.0	84	237.2	22.8	6.8	1.8 ± 1.0	0.2	52.7 ± 33.5	0.21 ± 0.03	8.0 ± 1.0	13.7 ± 3.8

DO: dissolved oxygen, OM: organic matter, DM: dry mass, phaeo: phaeopigments, WM: wet mass. Data are mean ± SE.

At each station, an USNEL Box corer (50 cm × 50 cm × 30 cm) was deployed for sediment collection (n = 1 per station due to time limitations at station). From each box core, three sub-cores of 10 cm diameter and approximately 20 cm sediment depth were taken for subsequent whole-core incubations. In addition, additional samples of upper 1 centimetre of sediment was collected outside the sub-cores with 10 mL truncated syringes (n = 3), for determining chlorophyll *a* (Chl *a*) and phaeopigment (phaeo) concentrations (mg m^−2^). These samples were immediately frozen at −80 °C for later processing. To determine organic matter content (% of dry sediment) and grain size distribution (%), 60 mL truncated syringes were used to collect the upper 5 centimetres of sediment (n = 1) and samples immediately frozen at –20 °C.

### Quantification of oxygen and nutrient fluxes (objective 1): sediment core incubations

After retrieval and preparation of the box corer on deck, sub-cores were pushed to ca. 15–20 cm into the sediment and taken immediately to a dark and temperature-controlled room (4 °C). Sediment cores were carefully topped with bottom water collected with the rosette at the same station. Incubation cores were acclimated for 6–8 h to allow recovery from sampling disturbance and the settling of suspended particles before the start of the incubations. During this period, the water was saturated with oxygen using an air aquarium pump to avoid suboxic conditions. Previous research has demonstrated that aeration of cores does not significantly alter the results or interpretation of sediment core incubation experiments [29]. After the acclimation period, sediment cores were sealed with a lid equipped with a magnetic stirrer and a sealable hole for water sampling and air bubbles were carefully removed. Sediment cores were incubated for 24–48 h until approximately 20% of available oxygen was consumed to minimize changes in oxygen availability during the incubation and to maintain comparable experimental conditions among cores. Three supplementary incubation cores, incubated with bottom water only, served as controls to assess oxygen uptake and nutrient changes due to processes in the water column or due to sample handling. To minimise the potential for spikes in photosynthetic activity, which could alter oxygen consumption within the cores, we used diffuse green light when inspecting the cores during the incubation period whenever necessary.

Oxygen concentration was measured continuously with oxygen sensor spots SP-PSt3-NAU (PreSens, Regensburg, Germany) attached on the inner wall of each incubation cores and a polymer optical fiber fixed on the outside of the incubation cores using a collar (Fibox 4, PreSens, Regensburg, Germany). To determine changes in nutrient concentrations (NH_4_^+^, NO_3_^−^, NO_2_^−^, Si(OH)_4_, PO_4_^3−^), water samples were collected at the beginning, midpoint and end of incubations (100%, 90% and 80% O_2_) and at the same time from the bottom water. Each time water was withdrawn from an incubation core for nutrient analyses, an equal volume of bottom water was added. A sample of this replacement water was collected and analyzed for nutrient concentrations, and these measured values were used to correct the calculated nutrient fluxes for the nutrients introduced during water replacement. To prevent spurious measurements, the volume of water withdrawn never exceeded 10% of the total volume of the incubation core. Water samples were filtered through 0.7 μm GF/F filters using acid-cleaned plastic syringes.

Measurements of nitrite, nitrate, phosphate and silicic acid were performed using a Bran and Luebbe Autoanalyzer 3 applying colorimetric methods adapted from Grasshoff et al. [30]. Additional water samples were analysed on board for ammonium measurements using a Turner Design fluorimeter following the method proposed by Holmes et al. [31]. The detection limit was 0.100 μmol L^−1^ for ammonium, 0.010 μmol L^−1^ for nitrate and nitrite, 0.020 μmol L^−1^ for phosphate and 0.016 μmol L^−1^ for silicic acid. The precision of these methods was ± 5%.

Based on the surface area of the cores and volume of water in the core tube, oxygen and nutrient fluxes were assessed from the slope of the linear regression of oxygen and nutrient concentration versus time of incubation and corrected for the solute concentration in the replacement water measured at each filling [32–34]. Median flux determined in the control core was subtracted from each sediment core measurement. A positive nutrient flux indicates a release of nutrients from the sediment into the water column, while a negative flux signifies an uptake of nutrients from the water column into the sediment.

### Environmental and diversity drivers of benthic ecosystem functioning (objective 2)

#### Macrofauna abundance, biomass and taxonomic diversity.

Following incubation, sediment cores were sieved through a 500 μm mesh sieve to collect infauna. Organisms were fixed with 4% buffered formalin solution and, in the home laboratory, sorted under a dissecting microscope and identified to the lowest possible taxonomic level with support of taxonomic experts. For each taxon, abundance (N; mean ± SE individuals), wet biomass (mean ± SE to the nearest mg) and taxonomic richness as the number of taxa present in each sediment core (mean ± SE) were determined. In addition, the Shannon-Wiener (H’) using the natural logarithm (ln) and Pielou’s evenness (J’) diversity indices were calculated for each sediment core using the “vegan” R package [35]. Taxa not identified to species level were distinguished from other species (e.g., as sp. 1) and classified as morpho-species. Where such consistency across the study region was not achievable, specimens were grouped into the lowest common taxon.

#### Biological traits and functional diversity.

Subsequently, species were classified into functional groups according to their traits in terms of feeding mode, body size, mobility and bioturbation influence (Table 2, Table S1). Traits were chosen based on their presumed influence on oxygen and nutrient fluxes. Biological traits were collected for each taxon from the Arctic Traits Database and other published sources [32,36–38]. Where biological traits information was unavailable for a specific taxon, we used information from one taxonomic rank higher. We used fuzzy coding that allowed more than one functional trait for a given taxon for each category and scored from 0 to 1 based on the extent to which they displayed each trait [39]. For example, the cumacean *Eudorellopsis integra* can alternate between deposit and filter feeding depending on environmental conditions, so these two traits each scored 0.5 for the feeding type category. If a taxon had 3 functional traits for a category, it would score 0.33 for each category.

**Table 2:** Categories of traits of Arctic benthic invertebrates and their levels (modalities) used to define functional groups.

Category	Level
**Feeding/diet**	C = Carnivorous/Predator
	D = Deposit feeder
	F = Filter/Suspension feeder
	O = Opportunist/Scavenger
	P = Parasite
**Size**	S < 3 mm
	3 mm < M < 10 mm
	L > 10 mm
**Mobility**	M = Mobile
	S = Sessile
	L = Limited
**Bioturbation**	B = Biodiffusor
	C = Up/down conveyor
	S = Surficial modifier
	N = None

Trait category scores for each taxon and taxa abundance matrices were used to obtain functional diversity (FD) indices using the “FD” package in R [40]. We then computed the following multidimensional FD indices for use in our analyses: functional richness (FRic), functional evenness (FEve) and functional divergence (FDiv) [40,41]. FRic represents the total functional space occupied by the community [41]. FEve, a unitless metric constrained between 0 and 1, describes the extent to which faunal abundances are evenly distributed within the trait space, where a value of 1 represents a perfectly even community [42]. FDiv quantifies heterogeneity of traits by representing the probability that 2 individuals picked at random from the community exhibit the same trait value [43].

We also determined community-level weighted means (CWMs) for each trait. CWM represents the probability that an individual organism drawn at random from the community will exhibit a given trait modality [42,44]. For example, a CWM of 0.6 for the trait modality ‘scavenger’ indicates a 60% probability that a random individual drawn from that community would be a scavenger.

### Sediment properties (objective 2)

Chlorophyll *a* and phaeopigment concentrations were quantified using a modified protocol by Riaux-Gobin and Klein [45] and Link et al. [46]. We placed 2 g of wet sediment from the top centimeter of the sediment in 10 mL of 90% acetone (v/v) for 24 h at 4 °C and then analyzed the supernatant before and after acidification (HCL 5%) using a TURNER Design 10AU fluorometer. Then, the sediment was dried and weighed to standardize pigment concentrations per gram of dry sediment. We sampled the top centimeter of the sediment sub-cores to quantify organic matter (OM) content by loss of ignition (500 °C). The water content was determined by comparing the mass of wet and dried sediment. We sampled the first five centimeters of sediment to determine granulometric properties (sediment mean grain size; MGS) with a Laser Diffraction Particle Size Analyzer LA-950 HORIBA. No sieving was performed prior to analysis because no large particles were present in our sediment samples.

### Sea ice cover

The presence and extent of sea ice cover at the sampling sites were determined from the Canadian Ice Service (https://iceweb1.cis.ec.gc.ca/Archive/page1.xhtml). A window of 2 weeks before the sampling with a concentration of ice less than 20% (i.e., from open water to very open drift) was considered as ice-free.

### Statistical analysis

#### Spatial variation of fluxes at the sediment-water interface (objective 1).

We used a mixed-model PERMANOVA design to test for spatial differences in fluxes between stations. The resemblance matrices quantifying between-replicate similarities for all six standardized fluxes (O_2_ and five nutrients) were calculated using Euclidean distances performed with 9999 random permutations. Missing data points were replaced using the ‘missing’ function in PRIMER-E software. This function attempts statistical estimation of a missing value by exploiting the correlation among variables to estimate the most likely value of a missing cell, thus completing the matrix. We verified homogeneity of multivariate dispersion using the PERMDISP routine [47]. We further analyzed significant terms in the full models using pair-wise comparisons. Non-metric Multidimensional Scaling (nMDS) plots were used to visualize the resemblance patterns. We completed PERMANOVA and PERMDISP analyses in PRIMER 6 with the PERMANOVA+ add-on [41].

#### Biogeochemical flux drivers (objective 2).

Two separate distance-based redundancy analysis (dbRDA) were performed using PRIMER-E software using the stepwise distance-based linear models permutation test (DistLM) to identify which subset of diversity and environmental variables best predicted the multivariate variation of six fluxes. For the DistLM analyses, taxonomic and functional diversity variables were combined into a single biological predictor set to evaluate the overall contribution of biodiversity relative to environmental variables. These two components were subsequently treated as separate explanatory groups in the variation partitioning analysis to quantify their independent and shared contributions. We determined the model with drivers that best explained variation in fluxes using a stepwise routine that employed 9999 permutations based on the AICc selection criterion. Draftsman’s plots of predictor variables indicated high correlation (r > 0.85) among some of our predictor variables including phaeopigment concentration and chl *a*:phaeo ratio, salinity and sea ice cover, and biomass, abundance and CWM of surficial modifiers. To reduce multicollinearity, we excluded phaeopigment concentration, salinity, biomass, and CWM of surficial modifiers. We retained the chl *a*:phaeo ratio because it better reflects the freshness of sedimentary organic matter than phaeopigment concentration alone; sea ice cover because it integrates the environmental conditions that structure primary production and organic matter supply; and abundance rather than biomass because biomass was disproportionately influenced by the presence of medium to large-bodied bivalves and dense aggregations of cumaceans at some stations. In addition, individual body size was already explicitly represented by the CWM body-size traits included in our analyses. The optimal environmental model selection included 8 variables: depth, sea ice cover, mean grain size, organic matter percentage, chl *a* concentration*,* chl *a*:phaeo ratio, bottom water temperature and dissolved O_2_ concentration. The optimal diversity model selection included 20 predictor variables: abundance, taxa richness, Shannon diversity, Pielou’s evenness, functional richness, functional evenness, functional divergence and the community weighted means of all respective traits except surficial modifiers and organisms with no bioturbation potential (Table 2).

To correct for data skewness, we applied a square root transformation to the predictor variables bottom water temperature, abundance and dissolved oxygen concentration. The response variables phosphate and ammonium required square root and cube root transformations, respectively. Nitrite required a natural log (ln) transformation. Prior to running the DistLM, we normalized flux, environmental and diversity data using the “normalise” function in PRIMER-E.

#### Variation partitioning of oxygen and nutrient fluxes with environmental, taxonomic and functional variables (objective 3).

Variation partitioning was used to determine relative contributions of environmental, taxonomic and functional diversity variables to flux variation. The method followed the principle used by Belley et al. [16]. Briefly, this was done by (1) performing a dbRDA of the flux on the environmental data, (2) performing a dbRDA of the flux on the taxonomic diversity data, (3) performing a dbRDA of the flux on the functional diversity data, (4) performing dbRDAs of the flux on these different subsets combined (environmental with functional, environmental with taxonomic and functional with taxonomic), (5) computing the adjusted R^2^ of the six dbRDAs, and (6) computing fractions of adjusted variation by subtraction.

## Results

### Taxonomic and functional structure of the benthic communities

We identified a total of 2213 specimens representing 66 different taxa in the sediments taken from the incubation cores, of which 19 were identified to genus and 12 to the species level (S1 Table). The most taxon rich phylum was Annelida, followed by Arthropoda*.* Taxonomic richness ranged from 4 taxa at RA25 (Kennedy Channel) to 24 at RA45 (Smith Sound) (Table 2). The lowest total abundances were found at RA25 and RA36 (4.3 ± 1.5 and 6.0 ± 0.0 individuals per core, respectively) and the highest were found in RA62 and RA54 in northern Baffin Bay (201.7 ± 21.0 and 329.3 ± 22.5) (Table 2). The most abundant class was Malacostraca. The most abundant species were two cumaceans, *Eudorellopsis integra* and *Brachydiastylis resima*, which were found in very high densities at stations RA54 and RA62. The second most abundant class was Polychaeta, which was also the most diverse. The most abundant Polychaeta species were *Prionospi steenstrupi* and *Laonice cirrata* (both Spionidae). Biomass ranged from 0.01 ± 0.01 g core^-1^ at RA25 to 0.35 ± 0.01 g core^-1^ at RA54 (Table 2). The highest biomasses recorded at stations RA54 and RA62 were largely attributable to the high abundances of the cumaceans *Eudorellopsis integra* and *Brachydiastylis resima.*

Functional richness ranged from 1.3 ± 0.0 at RA25 up to 16.6 ± 3.0 and 17.0 ± 2.2 at RA45 and RA38, respectively (Table 2). Functional evenness ranged from 0.5 ± 0.1 at RA54 to 0.9 ± 0.0 at RA36. And functional divergence from 0.8 ± 0.1 at RA38, RA45, RA54 AND RA58 to 0.9 ± 0.1 at RA36, RA50, RA62 and RA67 (S2 Table). Gradual changes in the CWMs between the northern and the southern stations were seen (Fig 2, S3 Table). Feeding trait composition shifted from a higher proportion of filter feeders and opportunistic/scavenger in the northernmost stations (RA25 and RA36) to a higher proportion of deposit feeders in the southern stations (RA38 to RA67). Spatial gradients were also evident for body size, mobility, and bioturbation potential. Northern stations were characterized by a higher prevalence of medium- to large-bodied, sessile taxa, whereas southern stations were dominated by small- to medium-bodied, mobile taxa with greater bioturbation potential (Fig 2, S3 Table).

**Fig 2:**
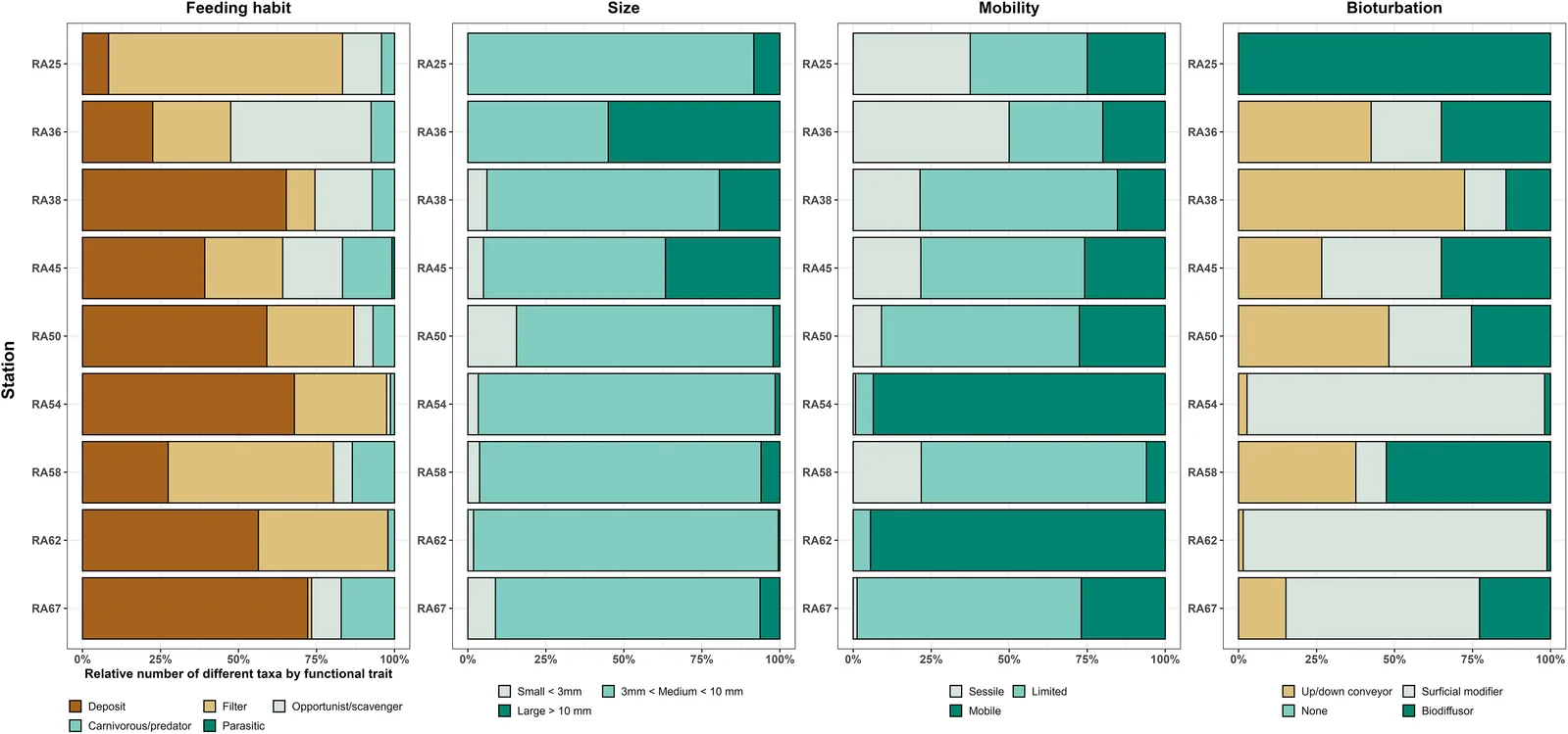
Functional trait composition of macrobenthic communities in Nares Strait in 2024.

### Spatial variability of fluxes

In most incubations, oxygen, nitrite and ammonium were taken up by the sediments, while nitrate, phosphate and silicic acid were mostly released, with an exception in northern Baffin Bay at station RA67 (Fig 3). Mean flux was lowest for all parameters in the north (RA25) and highest in the south (RA67; Fig 3). Sediment oxygen demand varied between 0.7 ± 0 and 4.4 ± 0.7 mmol O_2_ m^−2^ d^−1^ with the lowest values at the northernmost station in Kennedy Chanel (RA25) and the highest values at the central Kane Basin and northern Baffin Bay (RA38 and RA67; Fig 3a). In contrast, phosphate release (from 1.5 ± 1.0 to 61.2 ± 16.4 μmol PO_4_^3-^ m^−2^ d^−1^; Fig 3b), silicic acid release (from 1.8 ± 0.2 to 4.8 ± 0.9 mmol Si(OH)_4_ m^−2^ d^−1^; Fig 3c), and ammonium uptake (from 12.9 ± 7.4 to 106.3 ± 150.9 μmol NH_4_^+^ m^−2^ d^−1^; Fig 3d) exhibited different spatial patterns and did not show elevated fluxes in central Kane Basin (RA38). The patterns for nitrite and nitrate fluxes were more variable. While nitrite was taken up by the sediments at all stations (from 0.9 ± 1.3 to 5.5 ± 1.9 μmol NO_2_^-^ m^−2^ d^−1^; Fig 3e), it was released in RA67 (34.7 ± 19.9 μmol NO_2_^-^ m^−2^ d^−1^; Fig 3e). On the opposite, nitrate was taken up by the sediments in RA67 (78.8 ± 60.6 μmol NO_3_^-^ m^−2^ d^−1^; Fig 3f), while it was released in all the other stations (from 65.1 ± 13.8 to 331.6 ± 104.3 μmol NO_3_^-^ m^−2^ d^−1^; Fig 3f).

**Fig 3:**
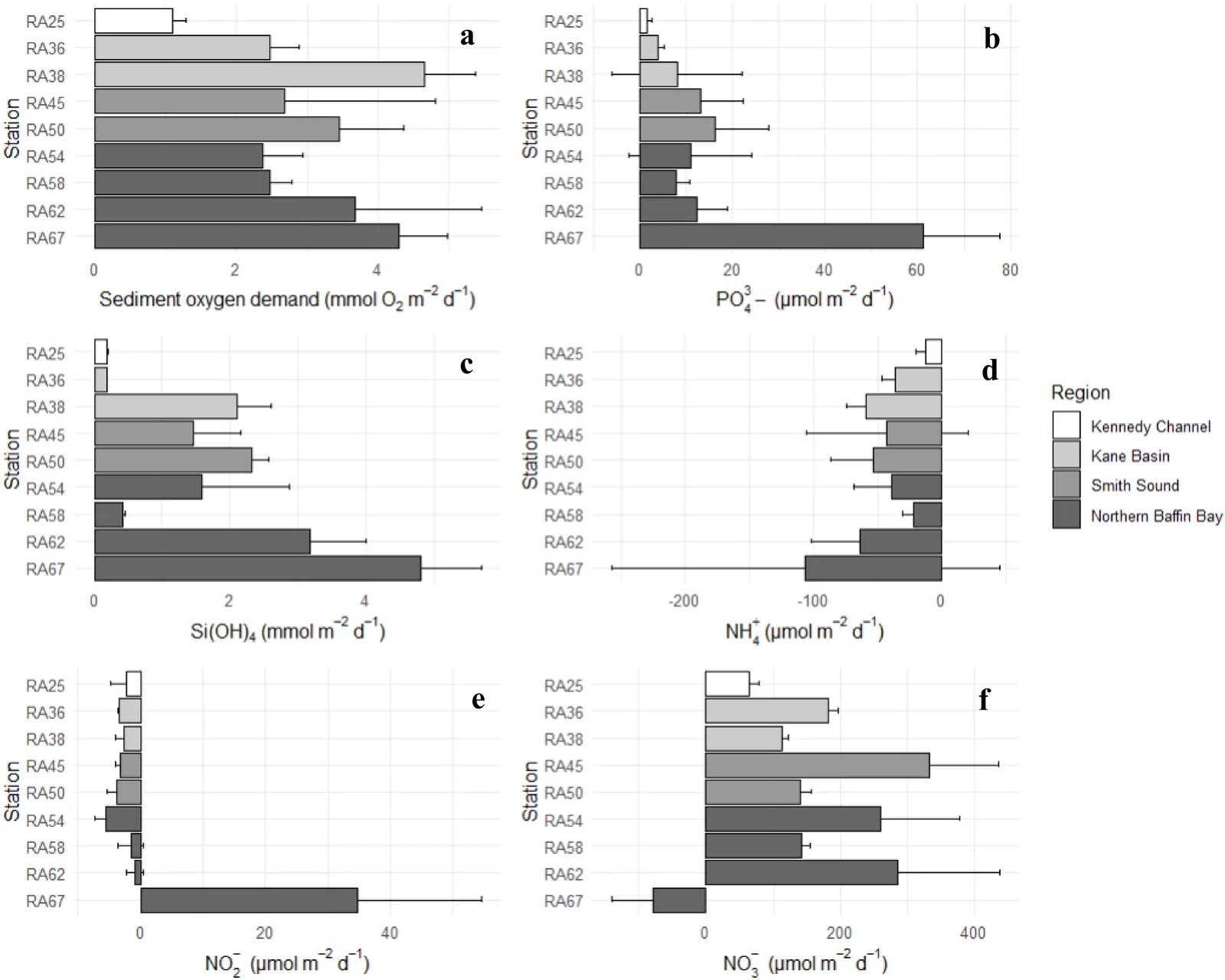
Mean (+SE, n = 3) of sediment-water interface flux of oxygen (a), phosphate (b), silicic acid (c), ammonium (d), nitrite (e) and nitrate (f) measured at each location. On the Y-axis are stations listed from RA67 in the south to RA25 in the north.

PERMANOVA indicated significant differences in flux between stations [P(perm) < 0.001]. PERMDISP indicated no significant differences in multivariate dispersion among stations (F = 1.85, p = 0.089). Although some stations exhibited larger average distances to the centroid than others (RA67, RA54 and RA62), the low number of replicates per station (n = 3) limits the statistical power of the test. Therefore, the significant PERMANOVA results are interpreted primarily as differences in fluxes among the stations rather than differences within-stations. Although a few pairwise comparisons were significant prior to correction for multiple testing, only the comparison between stations RA45 and RA62 remained significant after Holm/Bonferroni corrections (p-value = 0.036). Non-metric multidimensional scaling (nMDS) revealed a clear separation of stations based on oxygen and nutrient fluxes (stress = 0.11; Fig 4). The southwesternmost Station RA67 was clearly isolated from the remaining stations, yet with high variability among replicates. Replicates of the northernmost station RA25 formed a distinct cluster. In contrast, stations RA36, RA38, RA50 and RA58 largely overlapped, though Kane Basin station replicates RA36 and RA38 were all in the upper half of the plot while Smith Sound and NOW stations were spread throughout.

**Fig 4:**
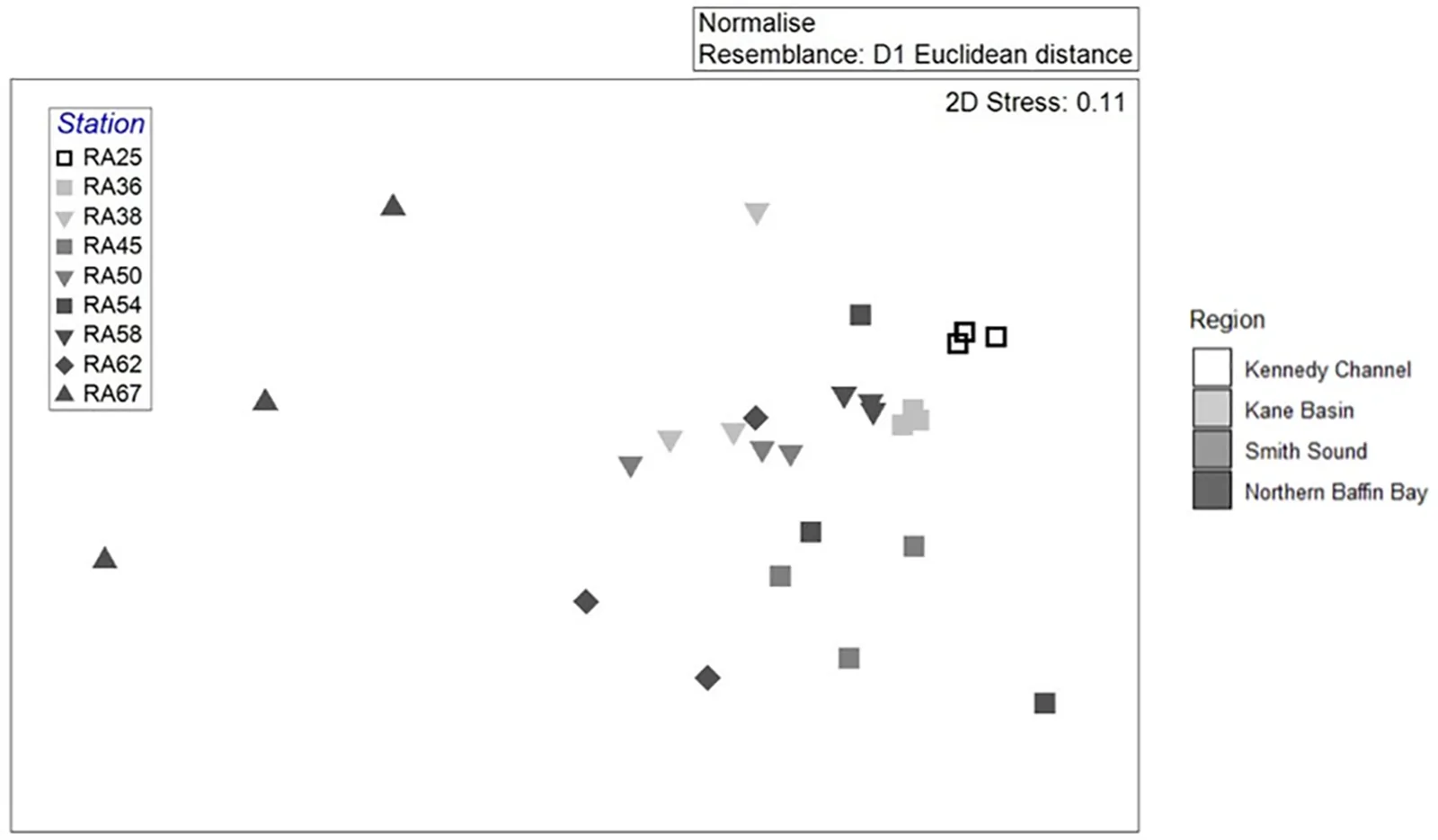
Non-metric multidimensional plot showing spatial pattern of benthic remineralization at each sampling station in Nares Strait in September 2024. The plot shows the relative distance of samples determined as Euclidean distance of the benthic biogeochemical fluxes. Each symbol represents one replicate.

### Drivers of spatial variability in biogeochemical fluxes

#### Sea ice cover, sediment grain size and food availability.

Chl *a* varied from 0.1 ± 0.0–0.1 μg g^−1^ DW at RA25 and RA36, respectively, to 2.9 ± 1.9 μg g^−1^ DW at RA58 (Table 1). Overall, sediments were coarser in Kennedy Channel, Kane Basin, and Smith Sound than in northern Baffin Bay, with mean grain size ranging from 35.6 μm at RA36 to 17.7 μm at RA58 (Table 1). OM content ranged from 1.4% at RA25 to 7.8% in RA36 with generally higher values in the southern part of the strait (Table 1). At the time of sampling, only RA25 and RA38 remained ice-covered. The remaining stations had been ice-free for 39–92 days, with the duration of open-water conditions varying among regions (Table 1).

The best distance-based linear model (distLM) between fluxes and environmental parameters explained 33.7% (R^2^ = 0.34, Adj. R^2^ = 0.25) of total flux variation and included 3 variables (Fig 5A). Sea ice cover (number of days since ice-free) contributed most to the variation (13.9%), followed by mean grain size (MGS; 11.1%) and depth (8.7%) (S4 Table).

**Fig 5:**
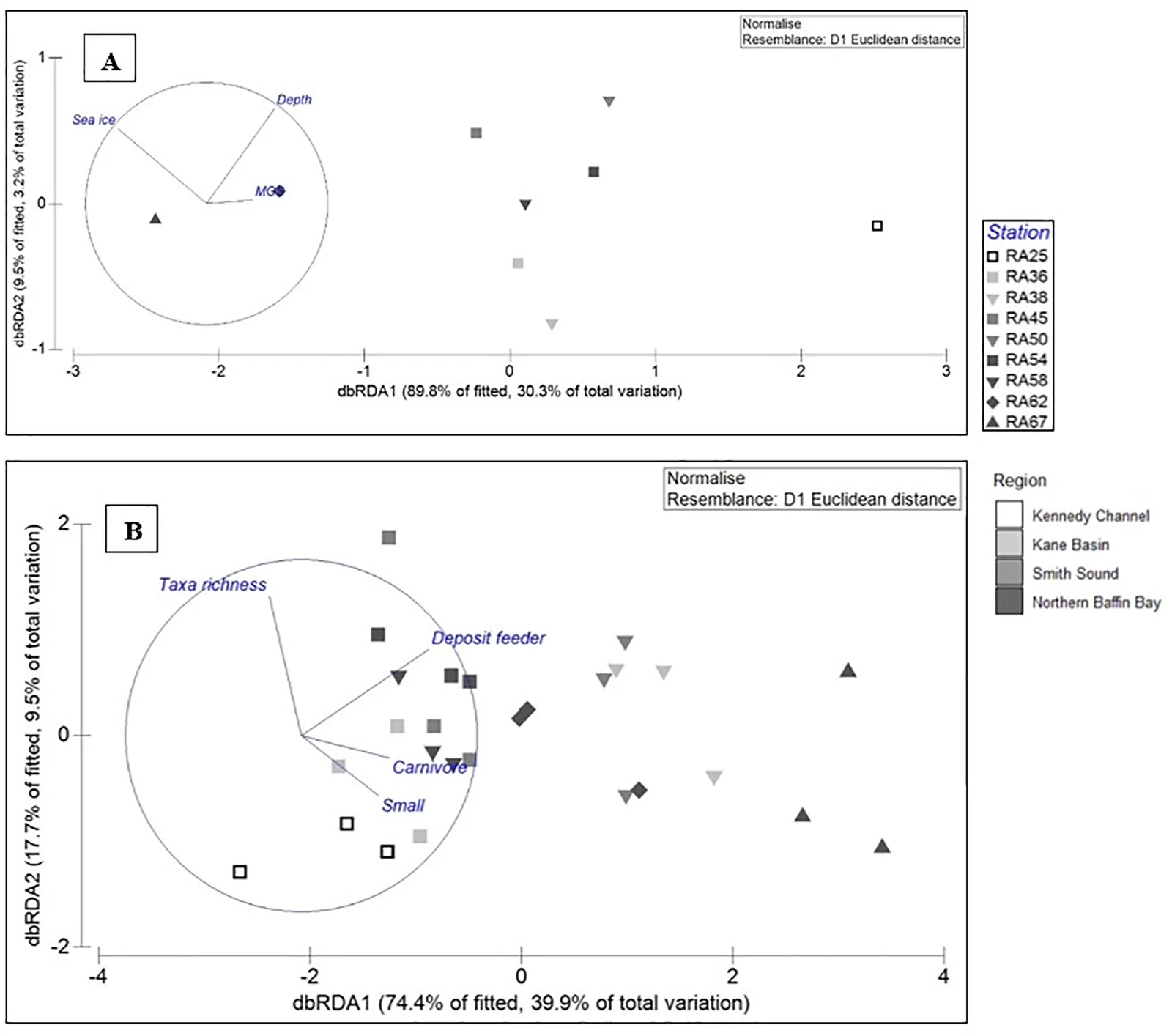
Distance-based redundancy analysis (dbRDA) plots of the distLM based on the environmental parameters (A) and the diversity indices (B) fitted to the variation in biogeochemical fluxes. MGS: mean grain size.

The first and the second axis account for 30.3 and 3.2% of the total flux variation, respectively. The most important parameters contributing to the first axis were sea ice cover and mean grain size, explaining 89.8% of fitted flux variation (Fig 5A, S4 Table). Depth was most strongly correlated with the second dbRDA axis, explaining 9.5% of the fitted flux variation.

#### Macrobenthic diversity.

The best distLM model between fluxes and macrobenthic taxonomic and functional diversity parameters explained 53.6% (R^2^ = 0.536, Adj. R^2^ = 0.452) of total flux variation and included 4 variables. CWM of deposit feeders explained the most variation (22.6%), followed by taxa richness (12.1%), CWM of carnivores (11.5%) and CWM of small organisms (7.4%) (S5 Table).

The first and the second axis account for 39.9 and 9.5% of the total flux variation, respectively. The most important parameters contributing to the first axis were CWM of deposit feeders, small organisms and carnivores, respectively (Fig 5B), explaining 74.4% of fitted flux variation. Taxa richness was most strongly correlated with the second axis, explaining 17.7% of fitted flux variation.

Overall, variation partitioning revealed that the three groups of predictors (environmental, taxonomic and functional) explained approximately 45% of the total variation in benthic biogeochemical fluxes, whereas 55% remained unexplained (Fig 6, S6 Table). Variation partitioning revealed that biogeochemical fluxes were to a larger degree explained by the shared contribution of environmental and macrobenthic functional variables (25% of the total variation explained) than by contributions from taxonomic (5%), functional or environmental variables (0%).

**Fig 6:**
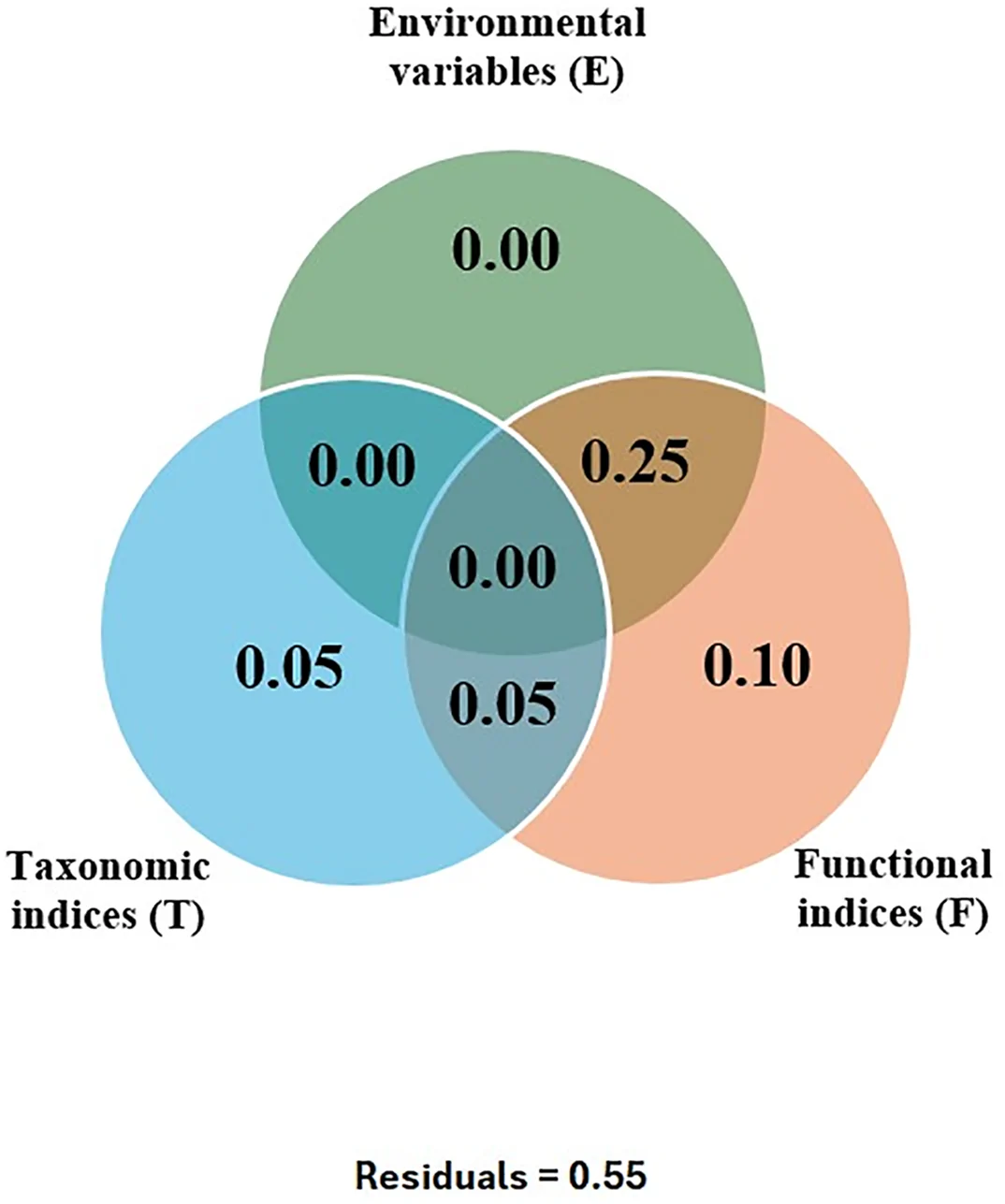
Venn diagram showing the variation partition of the environmental variables, taxonomic and functional diversity indices of macrobenthos in Nares Strait that contributes to explaining the sediment-water interface flux variation.

A further 5% of the variation was explained by taxonomic and functional descriptors. Similarly, no variation was exclusively shared between environmental and taxonomic variables and the three-way shared fraction among environmental, taxonomic, and functional descriptors was negligible.

## Discussion

The objective of this study was to identify the key drivers of spatial variability in oxygen and nutrient fluxes across the sediment-water interface in Nares Strait. Our results suggest a strong gradient in benthic functional diversity. The stations in Kennedy Chanel and northern Kane Basin were characterized by low abundances of larger, more sessile, suspension feeder and opportunistic taxa, compared to the stations in the central Kane Basin and northern Baffin Bay (in the NOW), which had a higher number of small and medium-sized, mobile, deposit-feeding taxa. Multivariate statistical analyses (i.e., dbRDA) allowed us to identify which subset of environmental, taxonomic or functional variables best explains fluxes variations. Overall, the three groups of predictors explained approximately 45% of the total variation in biogeochemical fluxes, leaving 55% of the variation unexplained. The presence of deposit feeders and sea ice cover (number of days since ice-free) explained the most variation in our models, accounting for 22.6% and 13.9% of flux variation, respectively. Additional drivers included taxa richness (12.1%), presence of carnivores (11.5%) and the mean grain size (11.1%). Variance partitioning revealed that functional indices were the best predictors of biogeochemical fluxes. The joint fraction between environmental and functional descriptors accounted for 25% of the total variation explained, representing the largest component of the model. Once functional descriptors were taken into account, environmental variables did not explain any unique variation, indicating that all information conveyed by environmental variables regarding biogeochemical fluxes is included within the functional structure of the benthic communities.

### Hydrography controls on sediment characteristics and associated redox conditions

Among the environmental variables measured, sea ice cover accounted for the largest proportion of explained variation in the environmental model (13.9%), followed by mean grain size (11.1%) and water depth (8.7%) (S4 Table). This finding is consistent with previous studies identifying sea ice dynamics as a major driver of Arctic benthic ecosystem functioning [8,48]. Although sea ice cover does not directly regulate oxygen and nutrient fluxes at the sediment-water interface, our results suggest that its influence is largely mediated through changes in sediment biogeochemistry. Together with mean grain size and water depth, sea ice conditions likely influence fluxes by regulating oxygen penetration depth and sediment redox conditions, thereby modifying the relative importance of the microbial pathways responsible for oxygen and nutrient cycling. Consequently, the environmental component of our models most likely reflects differences in local hydrographic and sea ice conditions that shape sediment characteristics and, ultimately, sediment redox environments.

These mechanisms are well illustrated by the pronounced environmental gradient observed across Nares Strait. Grain-size distribution followed a clear latitudinal pattern, with the coarsest sediments and lowest fluxes occurring in Kennedy Channel and Kane Basin, whereas the finest sediments and highest fluxes were found in northern Baffin Bay. This pattern likely reflects the strong hydrographic gradient characterizing Nares Strait, which has previously been shown to structure planktonic communities throughout the region [49]. The observed latitudinal variation in sediment grain size probably results from the combined influence of hydrodynamics, sea ice conditions, and sediment deposition. Strong tidal and throughflow currents in the narrow northern sections of the strait, particularly in Kennedy Channel, likely promote sediment winnowing and the preferential removal of fine particles, resulting in relatively coarse seabed sediments [50–52]. In contrast, northern Baffin Bay (where the NOW is located) functions as an important depositional environment where elevated particle export associated with high primary production favours the accumulation of fine terrigenous sediments and biogenic material [53,54]. Consequently, this region likely acts as a regional sink for fine particles transported through Nares Strait. Mean grain size therefore integrates the cumulative effects of hydrodynamics, sediment supply, and pelagic–benthic coupling operating across the sea ice gradient, explaining its strong relationship with oxygen and nutrient fluxes.

The fact that sea ice cover emerged as a significant predictor whereas sediment pigment concentrations did not suggest that benthic ecosystem functioning responded more strongly to long-term environmental conditions than to the instantaneous food supply measured at the time of sampling. Although food supply is widely recognized as a major driver of Arctic benthic ecosystem functioning [8], sediment pigment concentrations were not retained as significant predictors in our models. This likely reflects the timing of our sampling, which took place at the end of the productive season, i.e., sea ice in the strait was beginning to reform. Consequently, sediment pigments probably represented the residual organic matter pool remaining after the seasonal production peak rather than the magnitude of annual carbon delivery to the seafloor. This interpretation is supported by the low chl *a*:phaeopigment ratios indicating old, processed phytodetritus, suggesting that the sampling occurred months after the input peak. This is further supported by the relatively low oxygen uptake rates observed in our study (0.7–4.4 mmol m ⁻ ² d ⁻ ¹; < 660 m deep), which were comparable to those reported from the deep Arctic Fram Strait (0.5–5.1 mmol m ⁻ ² d ⁻ ¹; > 1500 m deep [48]) despite the substantially shallower depths and higher productivity characterizing parts of Nares Strait. Sea ice cover therefore likely acted as an integrative proxy for seasonal productivity, organic matter export, sedimentation regimes, and hydrographic conditions operating over longer temporal scales than the pigment concentrations measured during sampling.

### Benthic community effects on flux variation

Beyond the influence of environmental factors, benthic functional diversity plays a key role in regulating sediment biogeochemical processes. In our diversity models, the presence of deposit feeders explained an important part of flux variation (22.6%). However, the fact that the CWMs of deposit feeders explained more variation than the traits directly related to biorrigation or mobility suggests that deposit feeders do not explain flux variability only because they actively modify the sediment biogeochemistry. Rather, their distribution in Nares Strait seems to reflect long-term environmental conditions that also control fluxes, as previously discussed*.*

The strong relationship between deposit feeders and fluxes therefore likely arises because both respond to the same underlying environmental drivers. Indeed, the environmental variables that emerged as important drivers of fluxes (sea ice cover, grain size and depth) are precisely the factors known to structure deposit-feeding communities, and more generally benthic communities, throughout the Arctic [55–57]. Collectively, these environmental factors regulate the quantity, quality, and timing of organic matter reaching the seafloor, which is widely recognized as the primary determinant of the distribution and abundance of deposit-feeding communities in Arctic sediments [58,59]. Consequently, the strong association between deposit feeders and fluxes likely reflects their shared response to environmental conditions characterized by reduced sea ice cover, enhanced organic matter supply, and the accumulation of fine-grained sediments, such as those found in the North Water Polynya.

Taxa richness also emerged as an important predictor of flux variability, explaining 12.1% of the observed variation. Although higher biodiversity can directly enhance ecosystem functioning through niche complementarity and more efficient resource use, taxa richness in our study likely reflects the broader environmental conditions that support both diverse benthic communities and elevated biogeochemical activity. Indeed, taxa richness increased markedly along the north-south gradient, with the lowest values observed in Kennedy Channel and the highest in northern Baffin Bay. In Arctic ecosystems, areas receiving greater and more sustained inputs of organic matter often support higher benthic diversity while simultaneously exhibiting enhanced remineralization and nutrient regeneration rates, reflecting the strong coupling between food supply and benthic ecosystem functioning [56,60–62]. Consequently, taxa richness may act as an integrative indicator of habitat suitability and long-term food availability.

Interestingly, functional richness (FRic) had a low explanatory power in the flux variations relative to taxa richness. This result is consistent with previous findings from the NOW, where abundance and taxonomic diversity explained a larger proportion of flux variability than functional richness [32]. Previous studies have suggested that in ecosystems characterized by relatively low functional diversity, species richness can become a stronger predictor of ecosystem functioning because multiple species performing similar ecological roles increase functional redundancy [63]. This interpretation is consistent with our observations and suggests that species richness may provide a more sensitive indicator of benthic ecosystem functioning than functional richness in Nares Strait.

The importance of small-bodied organisms may reflect their rapid turnover rates and high biomass-specific metabolic activity. Consistent with metabolic scaling theory, smaller organisms generally process organic matter more rapidly than larger taxa and can respond quickly to variations in resource availability [64–66]. In line with this interpretation, a size gradient, although weak, was apparent along the strait. The stations in the Kennedy Channel and Kane Basin were characterized by a greater contribution from large organisms (> 10 mm) compared with the stations in the southern part of the strait (Fig 2). The prevalence of small-bodied taxa in southern Nares Strait may therefore reflect environmental conditions favorable to benthic processing, including enhanced organic matter deposition and fine-grained sediments, linking their distribution to the same hydrographic and productivity gradients driving fluxes. As with deposit feeders and taxa richness, the explanatory power of small-bodied organisms may primarily reflect their association with environmental conditions that promote elevated benthic activity.

Similarly, the contribution of carnivorous taxa to flux variability is unlikely to result from a direct influence on sediment biogeochemistry. Instead, carnivores likely act as indicators of ecosystem maturity and trophic complexity. Because higher trophic levels require sufficient prey availability and sustained energy transfer through the food web, greater abundances of carnivorous taxa are generally associated with productive environments supporting diverse and well-developed benthic assemblages [67]. Their relationship with sediment-water interface fluxes may therefore arise indirectly through their association with environmental conditions that promote both elevated organic matter supply and enhanced ecosystem functioning.

Overall, deposit feeders, carnivores, and small-bodied organisms may not primarily influence oxygen and nutrient fluxes through direct modification of sediment biogeochemistry. Rather, they appear to represent different biological manifestations of the hydrographic and productivity gradients operating across Nares Strait. This interpretation is supported by the variance partitioning analysis, in which the largest fraction of explained variation corresponded to the overlap between environmental and functional descriptors. Furthermore, environmental variables did not improve model performance once functional indices were included, suggesting that the influence of environmental conditions on sediment biogeochemistry is largely expressed through their effects on benthic community structure and functional composition.

### Unexplained variation in fluxes across the sediment-water interface

The variables included in this study accounted for approximately 45% of the observed variation in fluxes, leaving around 55% unexplained. This level of unexplained variation is not unexpected for benthic ecosystem processes, which result from the interaction of multiple environmental and biological mechanisms to measure simultaneously. In particular, our analyses did not account for processes occurring at finer spatial and temporal scales, such as microbial activity and meiofaunal processes. Among the environmental processes that may account for the unexplained variation, sediment redox dynamics represent an important candidate. Oxygen penetration depth (OPD), for example, integrates the balance between oxygen supply and consumption within the sediment and, as such, integrates the combined effects of sea ice conditions, organic matter supply, sediment characteristics, benthic activity, and microbial metabolism [1,68]. Consequently, differences in OPD may explain an important fraction of the flux variability that remained unresolved by the environmental and biological variables included in our models.

Evidence that sediment redox conditions may represent an important missing component comes from the nutrient patterns observed during the incubations. In particular, the consistent uptake of NH_4_^+^ coupled with substantial NO_3_^-^ release across stations (Fig 3) is indicative of active nitrification processes, which may have been enhanced by the oxygenation of the cores prior to the experiment. Prior to the incubations, the overlying water was gently aerated – as is the common protocol in these types of studies – to restore oxygen saturation close to 100%, a procedure that may have reduced the diffusive boundary layer and temporarily increased oxygen penetration into the sediments. Such conditions could have expanded the oxic layer and stimulated nitrification. However, previous studies have shown that comparable oxygenation procedures do not substantially alter the interpretation of sediment core incubations [29], suggesting that the observed response cannot be attributed solely to the experimental treatment. Rather, the results indicate that the sediments already possessed a strong potential for nitrification when oxygen availability increased.

The absence of a comparable response in sediments from this study conducted in the Salish Sea and Northeast Pacific, despite the use of similar experimental protocols, suggests that specific environmental and/or biological characteristics of Nares Strait sediments enhance their responsiveness to oxygenation. Compared with non-Arctic sediments, Nares Strait experiences strong seasonal pulses of organic matter deposition following sea ice breakup and primary production events. Such conditions may lead to elevated sediment oxygen demand, shallow oxygen penetration depths, and a compression of the nitrification zone near the sediment-water interface. Under these circumstances, nitrification may be constrained by oxygen availability, making it particularly sensitive to changes in OPD. In contrast, sediments from the Northeast Pacific may already possess deeper OPDs, reducing their sensitivity to additional oxygen supply.

Nevertheless, this explanation does not fully account for why similar experiments conducted in other Arctic regions did not stimulate nitrification to the same extent [32,36,69]. While the strong hydrographic gradient characterizing Nares Strait may generate more dynamic sediment redox conditions than those found in other Arctic shelf systems, additional biological factors may also contribute to the unexplained variation. In particular, microbial and meiofaunal communities were not included in our analyses despite their known influence on sediment biogeochemistry. Different microbial functional groups can strongly affect the rates and pathways of nitrogen cycling, including nitrification, denitrification, and anammox processes in Arctic sediments [70–72]. Similarly, growing evidence indicates that meiofauna (organisms <500 µm) can influence nutrient cycling indirectly through their effects on microbial activity and sediment microenvironments [73–75]. Consequently, differences in microbial and meiofaunal community composition may represent important missing drivers of flux variability. The strong hydrographic and productivity gradients operating across Nares Strait could select for communities with a greater capacity to exploit transient increases in oxygen availability, thereby enhancing nitrification following experimental oxygenation.

In addition, all incubations were performed at 4 °C because the temperature-controlled room available on board could not be set below this temperature, whereas in situ bottom-water temperatures ranged from −0.2 to 1.5 °C (Table 1). Although the higher incubation temperature may have enhanced microbial metabolism and faunal respiration, all stations were subjected to the same thermal conditions. Consequently, while absolute flux rates should be interpreted with caution, the relative differences among stations remain suitable for assessing the influence of environmental and biological gradients.

## Conclusion

This study demonstrates that oxygen and nutrient fluxes at the sediment-water interface in Nares Strait are primarily structured by large-scale hydrographic and sea ice gradients through their influence on sediment characteristics and benthic functional structure. Sea ice cover, mean grain size, and water depth emerged as the main environmental predictors of oxygen and nutrient flux variability across the sediment-water interface, highlighting the importance of long-term environmental forcing on benthic ecosystem functioning. Functional characteristics of benthic communities explained more variation in fluxes than environmental descriptors alone, indicating that benthic functional structure integrates the cumulative effects of environmental forcing and provides a powerful indicator of ecosystem functioning.

By encompassing one of the broadest gradients of sea ice and hydrographic conditions found in the Arctic, Nares Strait provides a natural laboratory for understanding how environmental change influences benthic ecosystem functioning. Because these conditions are representative of many Arctic marine systems, the relationships identified here likely extend beyond Nares Strait and provide new insight into how ongoing sea ice decline may reshape benthic ecosystem functioning and nutrient cycling across the Arctic Ocean. Building on these findings, a better understanding of the environmental drivers shaping benthic macrofaunal distribution and functional composition across Nares Strait will be essential for predicting how Arctic benthic ecosystems respond to continued climate-driven environmental change.

## Supporting information

S1 Table List of taxa identified in sediment cores and the accorded functional traits. For details on functional trait categories and levels, see Table 2.
(DOCX)

S2 Table Functional indices measured from the communities in the incubation cores. (DOCX)

S3 Table Community-level weighted means (CWMs) of each functional traits measured from the communities in the incubation cores. For details about functional traits, refer to Table 2.
(DOCX)

S4 Table Redundancy analysis of benthic fluxes against environmental drivers in Nares Strait in 2024. Mgs refers to mean grain size.
(DOCX)

S5 Table Redundancy analysis of benthic fluxes against diversity indices in Nares Strait in 2024. (DOCX)

S6 Table Results of the variation partitioning of the benthic fluxes between environmental parameters, taxonomic and functional diversity. (DOCX)
