# Supplementary material for: Hydrographic and sea ice gradients shape benthic ecosystem functioning through sediment characteristics and benthic functional structure in Nares Strait, Canadian Arctic
Source: PLoS One. 2026 Nov 13;21(11):e0345559. doi: 10.1371/journal.pone.0345559 (PMC13766610; doi:10.1371/journal.pone.0345559)
Supplement: Supplementary file 1 [file pone.0345559.s001.docx]

**Table S1.** **List of taxa identified in sediment cores and the accorded functional traits.** For details on functional trait categories and levels, see Table 2.

| **Class** | **Species/taxon** | **Feeding** | **Size** | **Mobility** | **Bioturbation** |
| --- | --- | --- | --- | --- | --- |
| Asteroidae |  | C | L | M | S/C |
| Bivalvia | *Bathyarca* sp. | F | M | S | S |
| Bivalvia | Cuspidariidae | C | M | L | B |
| Bivalvia | *Thyasira* sp. | F | M | L | C |
| Bivalvia | *Yoldiella* sp. | D | M | L | S |
| Clitellata | Oligochaeta | D | L | M | B |
| Echinoidea |  | D/C | L | M | S |
| Gastropoda | Acmaeidae | D | M | L | B |
| Gastropoda | Cancellariidae | C | M | L | S |
| Malacostraca | Ampeliscidae | F | M | L | S/C |
| Malacostraca | *Pseudosphyraphus anomalus* | D | S | L | S |
| Malacostraca | Apseudidae | D | S | L | S |
| Malacostraca | Calliopiidae | C | M | M | S |
| Malacostraca | Corophidae | F | M | L | S |
| Malacostraca | Desmosomatidae | D/O | S | M | B |
| Malacostraca | *Diastylis* sp. | D/F | M | M | S |
| Malacostraca | Diastylidae | D/F | M | M | S |
| Malacostraca | *Brachydiastylis resima* | D/F | M | M | S |
| Malacostraca | Gammaridae | D | S | M | B |
| Malacostraca | *Lamprops* sp. | D/F | M | M | S |
| Malacostraca | *Eudorellopsis integra* | D/F | M | M | S |
| Malacostraca | Leuconidae | D/F | M | M | S |
| Malacostraca | Munnidae | D | S | M | S |
| Malacostraca | Oedicerotidae | C | M | M | S |
| Malacostraca | Tanaidae | D | S | L | S |
| Ophiuroidea | Amphiuridae | D | L | M | S |
| Ophiuroidea |  | D/O | L | M | S |
| Ostracoda |  | D/C/O | S | M | S |
| Polychaeta | *Ampharete* sp. | D | M | L | C |
| Polychaeta | Capitellidae | D | S | L | C |
| Polychaeta | Cirratulidae | D | L | L | S |
| Polychaeta | *Chaetozone setosa* | D | M | L | S |
| Polychaeta | *Tharyx* sp. | D | M | L | S |
| Polychaeta | *Chaetozone* sp. | D | M | L | S |
| Polychaeta | *Cossura pygodactylata* | D | S | S | C |
| Polychaeta | *Clymenura* sp. | D | S | S | C |
| Polychaeta | *Cossura longocirrata* | D | M | L | S |
| Polychaeta | Cossuridae | D | M | L | S |
| Polychaeta | *Cossura* spp. | D | M | L | S |
| Polychaeta | Flabelligeridae sp. 1 | D | M | L | S/C |
| Polychaeta | Flabelligeridae sp. 2 | D | M | L | S/C |
| Polychaeta | Hesionidae | O | M | M | B |
| Polychaeta | Lumbrineridae | C/O | L | L/M | B |
| Polychaeta | Maldanidae | O | L | S | C |
| Polychaeta | *Clymenura polaris* | D | S | S | C |
| Polychaeta | *Aglaophamus* sp. | C/O | M | M | B |
| Polychaeta | Nephtyidae | C | S | M | C |
| Polychaeta | *Micronephtys minuta* | C | S | M | C |
| Polychaeta | Onuphidae | C/O | M | L | S |
| Polychaeta | Opheliidae sp. 1 | D | S/M | M | B |
| Polychaeta | Opheliidae sp. 2 | D | S/M | M | B |
| Polychaeta | Orbiniidae | D | M | L | B |
| Polychaeta | *Scoloplos* spp. | D | L | L | B |
| Polychaeta | Oweniidae | F/D | M | L | S |
| Polychaeta | *Cistenides hyperborea* | D | L | L | B |
| Polychaeta | Pectinariidae | D | M | L | C |
| Polychaeta | Phyllodocidae | C/O | M | M | B |
| Polychaeta | *Laonice cirrata* | D | M | L | C |
| Polychaeta | *Prionospio steenstrupi* | D | M | L | C |
| Polychaeta | Spionidae sp. 1 | D | M | L | C |
| Polychaeta | Spionidae sp. 2 | D | M | L | C |
| Polychaeta | Spionidae sp. 3 | D | M | L | C |
| Polychaeta | *Syllis cornuta* | C | M | M | B |
| Polychaeta | Syllidae | C | M | M | B |
| Polychaeta | Terebellidae | D | L | S | C |
| Anthozoa |  | C | M | L | S |
