# Supplementary material for: Hydrographic and sea ice gradients shape benthic ecosystem functioning through sediment characteristics and benthic functional structure in Nares Strait, Canadian Arctic
Source: PLoS One. 2026 Nov 13;21(11):e0345559. doi: 10.1371/journal.pone.0345559 (PMC13766610; doi:10.1371/journal.pone.0345559)
Supplement: Supplementary file 2 [file pone.0345559.s002.docx]

**Table S2. Functional indices measured from the communities in the incubation cores.**

| **Station** | **Functional richness** | **Functional evenness** | **Functional divergence** |
| --- | --- | --- | --- |
| **RA25** | 1.3 | 0.8 | 0.8 |
| **RA36** | 9.4 ± 2.9 | 0.9 ± 0.0 | 0.9 ± 0.1 |
| **RA38** | 17.0 ± 2.2 | 0.8 ± 0.0 | 0.8 ± 0.1 |
| **RA45** | 16.6 ± 3.0 | 0.7 ± 0.1 | 0.8 ± 0.1 |
| **RA50** | 11.0 ± 2.6 | 0.6 ± 0.1 | 0.9 ± 0.1 |
| **RA54** | 13.0 ± 3.7 | 0.5 ± 0.1 | 0.8 ± 0.1 |
| **RA58** | 16.3 ± 1.3 | 0.6 ± 0.0 | 0.8 ± 0.1 |
| **RA62** | 7.2 ± 2.1 | 0.6 ± 0.1 | 0.9 ± 0.1 |
| **RA67** | 13.7 ± 3.8 | 0.5 ± 0.2 | 0.9 ± 0.1 |
