# Supplementary material for: Hydrographic and sea ice gradients shape benthic ecosystem functioning through sediment characteristics and benthic functional structure in Nares Strait, Canadian Arctic
Source: PLoS One. 2026 Nov 13;21(11):e0345559. doi: 10.1371/journal.pone.0345559 (PMC13766610; doi:10.1371/journal.pone.0345559)
Supplement: Supplementary file 3 [file pone.0345559.s003.docx]

**Table S3. Community-level weighted means (CWMs) of each functional traits measured from the communities in the incubation cores.** For details about functional traits, refer to Table 2.

| **Station** | **Parasite** | **Carnivore/predator** | **Filter/suspension feeder** | **Opportunist/scavenger** | **Deposit feeder** | **Small** | **Medium** | **Large** |
| --- | --- | --- | --- | --- | --- | --- | --- | --- |
| **RA25** | 0.0 ± 0.0 | 0.0 ± 0.1 | 0.8 ± 0.3 | 0.1 ± 0.2 | 0.1 ± 0.1 | 0.0 ± 0.0 | 0.9 ± 0.1 | 0.0 ± 0.0 |
| **RA36** | 0.0 ± 0.0 | 0.1 ± 0.1 | 0.3 ± 0.1 | 0.4 ± 0.2 | 0.2 ± 0.2 | 0.0 ± 0.0 | 0.5 ± 0.1 | 0.7 ± 0.6 |
| **RA38** | 0.0 ± 0.0 | 0.1 ± 0.1 | 0.1 ± 0.0 | 0.2 ± 0.1 | 0.6 ± 0.1 | 0.0 ± 0.1 | 0.7 ± 0.1 | 0.0 ± 0.0 |
| **RA45** | 0.0 ± 0.0 | 0.1 ± 0.1 | 0.3 ± 0.1 | 0.2 ± 0.2 | 0.4 ± 0.1 | 0.0 ± 0.0 | 0.7 ± 0.3 | 0.3 ± 0.6 |
| **RA50** | 0.0 ± 0.0 | 0.1 ± 0.0 | 0.3 ± 0.1 | 0.1 ± 0.0 | 0.6 ± 0.1 | 0.1 ± 0.0 | 0.8 ± 0.1 | 0.0 ± 0.0 |
| **RA54** | 0.0 ± 0.0 | 0.0 ± 0.0 | 0.5 ± 0.0 | 0.0 ± 0.0 | 0.5 ± 0.0 | 0.0 ± 0.0 | 0.9 ± 0.0 | 0.0 ± 0.0 |
| **RA58** | 0.0 ± 0.0 | 0.0 ± 0.0 | 0.5 ± 0.0 | 0.1 ± 0.0 | 0.4 ± 0.0 | 0.0 ± 0.0 | 0.9 ± 0.0 | 0.0 ± 0.0 |
| **RA62** | 0.0 ± 0.0 | 0.0 ± 0.0 | 0.5 ± 0.0 | 0.0 ± 0.0 | 0.5 ± 0.0 | 0.0 ± 0.0 | 0.9 ± 0.0 | 0.0 ± 0.0 |
| **RA67** | 0.0 ± 0.0 | 0.2 ± 0.2 | 0.0 ± 0.0 | 0.1 ± 0.1 | 0.6 ± 0.2 | 0.1 ± 0.1 | 0.7 ± 0.2 | 0.0 ± 0.0 |

| **Station** | **Sessile** | **Limited** | **Mobile** | **Biodiffusor** | **Up/down conveyor** | **None** | **Surficial modifier** |
| --- | --- | --- | --- | --- | --- | --- | --- |
| **RA25** | 0.0 ± 0.0 | 1.0 ± 0.0 | 0.3 ± 0.6 | 1.0 ± 0.0 | 0.0 ± 0.0 | 0.0 ± 0.0 | 0.0 ± 0.0 |
| **RA36** | 0.3 ± 0.6 | 0.3 ± 0.6 | 0.0 ± 0.0 | 0.3 ± 0.6 | 0.4 ± 0.2 | 0.0 ± 0.0 | 0.2 ± 0.1 |
| **RA38** | 0.0 ± 0.0 | 0.7 ± 0.6 | 0.0 ± 0.0 | 0.0 ± 0.0 | 0.7 ± 0.1 | 0.0 ± 0.0 | 0.1 ± 0.1 |
| **RA45** | 0.0 ± 0.0 | 0.7 ± 0.6 | 0.0 ± 0.0 | 0.0 ± 0.0 | 0.3 ± 0.2 | 0.0 ± 0.0 | 0.4 ± 0.1 |
| **RA50** | 0.0 ± 0.0 | 1.0 ± 0.0 | 0.0 ± 0.0 | 0.0 ± 0.0 | 0.5 ± 0.1 | 0.0 ± 0.0 | 0.2 ± 0.1 |
| **RA54** | 0.0 ± 0.0 | 0.0 ± 0.0 | 1.0 ± 0.0 | 0.0 ± 0.0 | 0.0 ± 0.0 | 0.0 ± 0.0 | 1.0 ± 0.0 |
| **RA58** | 0.0 ± 0.0 | 1.0 ± 0.0 | 0.0 ± 0.0 | 0.7 ± 0.6 | 0.4 ± 0.0 | 0.0 ± 0.0 | 0.1 ± 0.0 |
| **RA62** | 0.0 ± 0.0 | 0.0 ± 0.0 | 1.0 ± 0.0 | 0.0 ± 0.0 | 0.0 ± 0.0 | 0.0 ± 0.0 | 1.0 ± 0.0 |
| **RA67** | 0.0 ± 0.0 | 0.3 ± 0.6 | 0.3 ± 0.6 | 0.0 ± 0.0 | 0.3 ± 0.2 | 0.0 ± 0.0 | 0.5 ± 0.3 |
