# Supplementary material for: Hydrographic and sea ice gradients shape benthic ecosystem functioning through sediment characteristics and benthic functional structure in Nares Strait, Canadian Arctic
Source: PLoS One. 2026 Nov 13;21(11):e0345559. doi: 10.1371/journal.pone.0345559 (PMC13766610; doi:10.1371/journal.pone.0345559)
Supplement: Supplementary file 4 [file pone.0345559.s004.docx]

**Table S4**. **Redundancy analysis of benthic fluxes against environmental drivers in Nares Strait in 2024.** Mgs refers to mean grain size.

| SEQUENTIAL TESTS | |  |  |  |  |  |  |
| --- | --- | --- | --- | --- | --- | --- | --- |
| Variable | AICc | SS(trace) | Pseudo-F | P | Prop. | Cumul. | res.df |
| +ice | 47.827 | 21.636 | 4.0256 | 0.004 | 0.13869 | 0.13869 | 25 |
| +depth | 47.487 | 13.611 | 2.7052 | 0.053 | 8.7248E-2 | 0.22594 | 24 |
| +mgs | 46.072 | 17.358 | 3.8612 | 0.004 | 0.11127 | 0.33721 | 23 |

| BEST SOLUTION | |  |  |  |
| --- | --- | --- | --- | --- |
| AICc | R^2^ | RSS | No. Vars | Selections |
| 46.072 | 0.33721 | 103.4 | 3 | 1-3 |
