# Supplementary material for: Hydrographic and sea ice gradients shape benthic ecosystem functioning through sediment characteristics and benthic functional structure in Nares Strait, Canadian Arctic
Source: PLoS One. 2026 Nov 13;21(11):e0345559. doi: 10.1371/journal.pone.0345559 (PMC13766610; doi:10.1371/journal.pone.0345559)
Supplement: Supplementary file 5 [file pone.0345559.s005.docx]

**Table S5**. **Redundancy analysis of benthic fluxes against diversity indices in Nares Strait in 2024.**

| SEQUENTIAL TESTS |  |  |  |  |  |  |  |
| --- | --- | --- | --- | --- | --- | --- | --- |
| Variable | AICc | SS(trace) | Pseudo-F | P | Prop. | Cumul. | res.df |
| +CWM.Deposit.feeder | 44.939 | 35.269 | 7.3033 | 0.001 | 0.22608 | 0.22608 | 25 |
| +sqrt_CWM_carnivore | 43.147 | 17.909 | 4.1802 | 0.005 | 0.1148 | 0.34089 | 24 |
| +taxarichness | 40.446 | 18.874 | 5.1711 | 0.001 | 0.12099 | 0.46187 | 23 |
| +sqrt_CWM_small | 39.482 | 11.568 | 3.5159 | 0.007 | 7.4151E-2 | 0.53602 | 22 |

| BEST SOLUTION | |  |  |  |
| --- | --- | --- | --- | --- |
| AICc | R^2^ | RSS | No. Vars | Selections |
| 39.482 | 0.53602 | 72.38 | 4 | 2,9,12,13 |
