# Supplementary material for: Hydrographic and sea ice gradients shape benthic ecosystem functioning through sediment characteristics and benthic functional structure in Nares Strait, Canadian Arctic
Source: PLoS One. 2026 Nov 13;21(11):e0345559. doi: 10.1371/journal.pone.0345559 (PMC13766610; doi:10.1371/journal.pone.0345559)
Supplement: Supplementary file 6 [file pone.0345559.s006.docx]

**Table S6**. **Results of the variation partitioning of the benthic fluxes between environmental, taxonomic and functional diversity.**

| Environmental (E), taxonomic (T) and functional (F) | Df | R.squared | Adj.R.Squared |
| --- | --- | --- | --- |
| E | 3 | 0,337 | 0,251 |
| T | 1 | 0,084 | 0,048 |
| F | 3 | 0,423 | 0,348 |
| E+F | 3 | 0,474 | 0,404 |
| E+T | 6 | 0,423 | 0,348 |
| T+F | 4 | 0,536 | 0,452 |
| E+T+F | 4 | 0,536 | 0,452 |
| Individual fractions |  |  |  |
| E unique |  |  | 0,000 |
| T unique |  |  | 0,048 |
| F unique |  |  | 0,103 |
| E ∩ T |  |  | 0,000 |
| E ∩ F |  |  | 0,251 |
| T ∩ F |  |  | 0,050 |
| E ∩ T ∩ F |  |  | 0,000 |
| Residuals |  |  | 0,548 |
