# Supplementary material for: Hydrographic and sea ice gradients shape benthic ecosystem functioning through sediment characteristics and benthic functional structure in Nares Strait, Canadian Arctic
Source: PLoS One. 2026 Nov 13;21(11):e0345559. doi: 10.1371/journal.pone.0345559 (PMC13766610; doi:10.1371/journal.pone.0345559)
Supplement: Supplementary file 7 [file pone.0345559.s007.docx]

Review Combaz et al.

The title is quite wordy. Maybe consider rephrasing. In particular “a new perspective through the lens of” is not really saying much. Also, “Importance of” in the beginning is rather unprecise.

I don’t really understand the use of the “contributed equally” symbols. Maybe you mixed this up.

¶These authors contributed equally to this work. This symbol is only placed behind the first author

&These authors also contributed equally to this work. This symbol is used for all other authors.

L27-31 These two introductory sentences sound a bit awkward. Maybe rephrasing the first sentence to already introduce a bit more specific the idea of different remineralization under different sea ice regimes could be considered.

L31 you incubated sediment cores in different location (in situ?) or you incubated cores collected from different locations (ex situ?)?

L32 different… different. Rephrase to avoid repeating the word

L33 If you measured oxygen and nutrients, maybe you could emphasize nutrient cycling in your opening sentence and not also carbon. Sure, some aspects of dissolved carbon fluxes are linked to consumption of oxygen, but you have many remineralization processes that do not use O2 as terminal electron acceptor. So you rather study oxygen consumption and not carbon cycling.

L49 Same as in the abstract. I don’t think carbon storage and cycling is really the topic you are studying.

L50-52 This is imprecise and not correct as stated. Benthic communities don’t directly remove CO₂ from the atmosphere. They process organic carbon delivered from the water column. Also, “removing carbon” implies sequestration or burial. Recycling is quite the opposite of removal.

L58-59 “Yet, the drivers of benthic remineralization are still poorly understood on regional scales.” I think this is not correct. Sure there is much that we do not know, but you do not provide any information about the drivers of remineralization. At least some mentioning of the redoxcline and the principles of the biogeochemical processes involved here should be mentioned. Organic matter quality, temperature, grainsize/porosity, bioturbation & bioirrigation. There is much we do know.

L60 benthic biodiversity? Also substrate type is very important for benthic communities. I am not sure depth itself is a particularly important driver itself. It’s rather a proxy of the other drivers that change with depth.

L64-65 I am not sure these studies are so recent anymore. They are all at least 10 years old.

L63-64 can you provide some references for such historic studies?

L63-69 Can you be a bit more precise here? Sure functional traits are mainly defined to describe ecosystem functions. But what traits and modalities are relevant for what processes. And which relate actually to your study objective (remineralization and fluxes)?

L69 same as before. 2008 or 2010 are not really recently.

L70-71 Isn’t this contradicting your previous statement that drivers are poorly understood on a regional scale?

L79 carbo…. N is missing.

L80-89 Please refer to your map where the different locations can be looked up.

L89-92 I don’t think your introduction really fits to your objectives. First, you hardly mention nutrients in your introduction. Why are they actually important and why would you want to study them. Second, why are you studying oxygen uptake? You haven’t mentioned oxygen so far. Third, you describe different ice conditions in the different subregion, but you never establish that they might be associated with different production and OM export regimes. Fourth, evaluating potential environmental drivers is fine, but you haven’t established based on literature what environmental drivers are important in other studies. Fifth, it’s not one process, it’s processes. It would be useful to also describe the different nutrients you consider and key components of their cycling.

Besides, you do not study the fluxes of the benthic communities, you study the fluxes across the sediment water interface.

L95-113 The study site description should be improved. One aspect is the language which should be improved, but also the content. Please adjust this to your study objective. You do not mention anything about benthic habitats, OM export, or productivity. Maybe bottom currents?

Table 1 Didn’t you write before that it is about 500m deep? Some of your stations are much deeper. Also you are inconsistent in using “North Water Polynya” and “Northern Baffin Bay”. This should be at least clarified. I am not sure that longitude should be reported with – if you also write that it is degree W.

Overall, I find you are reporting some numbers with too many decimal places in table 1

L128 it’s not nutrient remineralization… you look at nutrient fluxes. You cannot know if remineralization is ongoing during your experiments or if just the dissolved nutrients in the porewater are released.

L128-131 you did all of this with one small syringe sample? I guess not, so please correct the text. Besides, I find the sysringe size and sampling depth of 1 cm appropriate for pigment samples. But for OM content and grain size, I think larger volumes and deeper penetration depth should be used. Also, if you did 1 replicate, what are the numbers between the +- sign showing?

L134-137 maybe field sampling is the wrong section for this?

L139-140 I find this very limited for actually describing your core variables you are trying to explain. You measure all these variables, but they all are linked to different processes: e.g. respiration, nitrogen cycle processes, phosphorus regeneration or dissolution of biogenic Silica. Not all of these fluxes are necessarily indicating OM remineralization. Phosphate can be released from iron bound P. Dissolved Silica comes primarily from dissolution of biogenic silica. For example diatom frustules. These frustules are not OM themselves.

L140-141 Why is this here? Isn’t the next section about the fluxes?

L143-144 Are these the same cores you mentioned in L126-8? Or are they different?

L144 I understand that you had probably limited possibilities to reduce the temperature below 4 degree, but according to table 1, you study sites have bottom temperatures were between -0.2 and 1.5 degrees. Considering the great depth, the fauna and microbes in your cores are very unlikely to ever experience such high temperatures in their natural environment. This is a very important limitation of your experiment and needs to be considered carefully when interpreting your results.

L146 is it really for settling of particles or rather for the fauna to behave more normally after the stress of being collected.

L147-151 I understand that this is pretty standard procedure for these experiments, but according to table 1 the DO you measured in-situ was not so high. At your temperature the 240uM that you have on average at your stations is probably around 70% saturation. So by oxygenating your cores to 100%, you add oxygen in comparison to in-situ. And then you even stop your experiment at 80% saying that below this the biogeochemistry might change. But if in-situ oxygen levels are lower than this, then your argument would suggest that your experiment was conducted under conditions that do not reflect the in-situ biogeochemical processes.

L156-158 You are not measuring oxygen flux, you measure oxygen concentration and then you calculate the flux from the change in concentration. Did you really measure continuously? Or rather frequently? I assume you calibrated the optode before the cruise?

L159-160 how did you deal with replicate cores consuming O2 at different rates? Did you still collect samples from every core of the experiment at the same time? OR did you do this individually for each core? And what about your control cores with only bottom water?

L161-162 what do you mean with known solute concentration? Did you collect a sample of the bottom water for nutrient analysis everytime you replaced some water in your cores?

L164 Have you tested whether using GF/F filters actually affects your dissolved silica concentrations?

L166 So ammonium was measured from a different sample than the other nutrients?

L165-169 please provide some information on the used standards and measurement error.

L170-172 You also need to correct for the water volume in your core. You wrote the sediment cores were 15-20cm long. This makes quite a difference and needs to be corrected for after you corrected for your control cores.

L183 what base for the logarithm?

L190 I agree in general with the traits you selected, but you should justify why you consider each of them relevant. Maybe already in the introduction.

L222 2 taxa or 2 individuals?

L235 why just the top centimeter? How does this relate to the depth were your remineralization processes are happening and your fauna is active?

L243-272 please be more precise in how you describe your data analysis. I am not completely able to follow and would not be able to repeat your analysis.

L246 and what does the ‘missing’ function do?

L248 appropriate pair-wise comparison?

L253-256 What are these different subsets that you used in the separate analyses?

L256 why 5? Didn’t you have oxygen and 5 nutrients? That would be 6.

L259 you need to also provide information on what variables they correlated with. Otherwise it is impossible to interpret your results correctly. You are making an arbitrary choice in what you exclude.

L275 These general pattern suggests that you have probably altered redox conditions in the sediment through the oxygenation of the water in your cores. In particular that NH4+ was taken up by the sediments across all your experiments and that in parallel NO3- was released in large amounts is almost certainly partly caused by your sample treatment. I will explain. As I mentioned above, you started with not fully saturated bottom water (in-situ). Your oxygenation of the cores increases the saturation (typically close to 100%) and also reduces the diffusive boundary layer. Consequently, oxygen is penetrating deeper into your sediments, which activates nitrifiers. So the pattern you see is indicating that you artificially increased nitrification (i.e. NH4+ is consumed and NO3- produced). This is not completely invalidating your experiments, but you need to consider this carefully when interpreting your data.

Also, this highlights a key problem in the story you are telling. It’s not all remineralization! Fluxes also reflect other biogeochemical processes, like nitrogen transformation.

L276 Did you introduce this abbreviation? What does NOW stand for?

L277&281 I don’t understand why you present oxygen in mmol and SiO2 in micromole. They span almost exactly the same range of values, but one is now given as max 4.4 mmol and the other one as max 4800 micromol.

L279-282 That’s not correct, your figure 2 does not show the same pattern for O2, PO43-, DSi, and NH4+.

L281 It’s actually more appropriate to report the Silica as DSi and not SiO2. The molybdate blue method measures dissolved, reactive silica (DSi) and this is also the form in which the fluxes occur. Writing SiO2 is giving the impression that your fluxes would be in solid mineral form.

L283-286 This indicates that at RA67 you had incomplete nitrification.

L292-3 This statement is not correct. PERMANOVA tests for differences in group means. It’s not testing variability.

L294 PERMDISP is addressing variability, but only to test whether dispersion (variability) differed between the different groups. Not comparing within vs among experiment variation. Your previous statement is still not accurate according to your tests, but in general the PERMDISP supports that permanova result is not related to differences in dispersion. Anyhow, please keep in mind that your PERMDISP has very low power due to the low number of replication in each experiment (n=3).

L296 For the pairwise comparisons you should apply a p-value correction for multiple testing. With alpha = 0.05, you have to expect that on average you have 2 false positives for the 36 pairwise comparisons you are running to compare all your experiments.

L298-299 NMDS is a visualization of a dismilarity matrix. It does not test for differences in across and within site variability. It’s correct that station RA67 is very different from all the other. You could make this already clear when you describe Figure 2. Maybe for the nMDS you could consider excluding this station. It seems like this difference is so dominant that you might miss patterns in the remainder of your experiment.

L331-334 I don’t really understand why you are not also considering the environmental variables as another set in the variation partitioning. And similarly, why you did not separate functional from taxonomic diversity in the distLM before.

Besides, I don’t follow the description in the methods completely, but it sounded like you had a very small set of taxonomic diversity measures that you considered (Shanon, Pielou, taxon richness). But for functional diversity you use the three diversity measures and the CWM for all 16 modalities. So you consider 19 functional variables… This is very unbalanced and it’s obvious that this is largely causing your difference between functional and taxonomic diversity.

Overall, what I missed completely from the result section is a description of the benthic communities (taxonomic and functional). After reading your paper I do not have any idea what benthic communities were in your cores.

In general, the discussion lacks any consideration of biogeochemistry, which is extremely limiting. Also, for much of the discussion it is not really clear to me what the authors want to achieve with what they wrote. Much of what appears here should have probably been parted of the introduction and not written in the discussion where it is only loosely related to your results.

Since the issues pointed out above are expected to change the results, I am providing less detailed comments on the discussion.

L347 how did this stand out. You just wrote that in other studies the same range was observed.

L358 but RA36 had the same low Chl a.

L362-363 Even if these should be all available studies, you refer to 11 studies that’s not so little information.

L372-373 but in table 1, this station is not outstanding high in pigments and organic matter.

L379 To be quite honest, you need to consider biogeochemistry here. As I wrote above, you seem to have incomplete nitrification here. Also you have strong PO43- release. Both indicate a more shallow oxygen penetration in the sediments in this experiment than in the other experiments.

L380-384 finally some mentioning of other processes. You should look more into biogeochemical processes and discuss your patterns against them.

L387 To be honest, you have never given any justification for why all of this should function differently in the Arctic than anywhere else.

L387-389 REDOX conditions in the sediment!!!

L446-450 Ok, but how is this related to your study. You have not shown ice algal distribution in your study. Also, how would this then result in your fluxes. The deposit feeders would excrete nitrogen in the form of NH4+. But your sediments did take up NH4+.

L455-457 but in ice free systems, you also have deposit feeder dominating at similar depth. What other feeding modes would you realistically expect to dominate down there?

L463-466 This argument doesn’t make sense. You have bioturbation mode as a trait. Why would carnivory and scavenging appear as placeholders for bioturbation?

L473-475 These are so important because their burrows ventilate the sediment… REDOX conditions
