# Supplementary material for: Hydrographic and sea ice gradients shape benthic ecosystem functioning through sediment characteristics and benthic functional structure in Nares Strait, Canadian Arctic
Source: PLoS One. 2026 Nov 13;21(11):e0345559. doi: 10.1371/journal.pone.0345559 (PMC13766610; doi:10.1371/journal.pone.0345559)
Supplement: Supplementary file 8 [file pone.0345559.s009.docx]

**Response to the General Editor**

Dear Editor and reviewers, thank you for the careful assessment of our manuscript and for identifying these points. We have addressed all of the requested revisions as follows:

**(Please note that all page and line numbers mentioned are with the track-changes on).**

1. PLOS ONE formatting and file naming
We have revised the manuscript and associated files to ensure that they comply with the PLOS ONE formatting and file-naming requirements.

2. Fieldwork permits
We have added the relevant permit information to the Methods section, including the full name of the authority responsible for approving access to the field site and permit number (page 11, line 235).

3. Funding information
We have reviewed and corrected the funding information to ensure that the grant numbers reported in the Funding Information and Financial Disclosure sections are consistent and accurate.

4. Data availability
We have deposited the minimal dataset required to reproduce the results of this study in figshare. The Data Availability Statement has been updated accordingly and now includes the relevant doi: https://doi.org/10.6084/m9.figshare.33277044.

5. Copyright of Figure 1
We have replaced the map in Figure 1 with a new version using only public data. The figure caption has also been updated to provide the appropriate source and licensing information (page 9, line 199).

**Reviewer #1**

**General comment**: Dear authors,
I consider your study topic in general interesting and timely, but I find that there are too many short comings that needs to be addressed. Some methodological problems with your experiments that you need to carefully consider. This is not invalidating your results but it needs to be addressed openly throughout the manuscript since it affects interpretation.

**Response:** We thank the reviewer for this thoughtful and constructive assessment of our work. We appreciate the recognition that the study addresses an interesting and timely topic.

We agree that the methodological limitations should be discussed more explicitly so that readers can properly interpret our findings. In the revised manuscript, we have substantially expanded the discussion of the experimental limitations. In particular, we now explicitly discuss the effects of the initial oxygenation of the sediment cores, the implications of laboratory incubations for natural sediment processes, and how these factors may have influenced the absolute magnitude of the measured benthic fluxes. We also clarify that these methodological constraints do not preclude comparisons among stations because all incubations were conducted under the same experimental conditions. These limitations are now discussed in the methods (page 15, lines 295) and their potential effects addressed in the discussion (page 38-40, lines 810-863)

Comment: Actually the biggest problem with your manuscript in my opinion is that you study faunal influence on biogeochemistry, without consideration of biogeochemical processes. This doesn't work.

Response:

To address this concern, we have substantially revised the introduction and discussion to better integrate the sediment biogeochemical framework of our study. We now describe how benthic oxygen and nutrient fluxes emerge from the balance between oxygen supply, organic matter mineralization, microbial metabolism, and faunal activities such as bioturbation and bioirrigation. We also expanded the discussion of oxygen penetration depth and sediment redox conditions as key integrative processes linking environmental conditions, benthic communities, and fluxes. Finally, we clarify that the identified functional traits should be interpreted as indicators acting within this broader biogeochemical context rather than as independent drivers of benthic fluxes.

**Specific comments**

**Comment:** L27-31 These two introductory sentences sound a bit awkward. Maybe rephrasing the first sentence to already introduce a bit more specific the idea of different remineralization under different sea ice regimes could be considered.

**Response:** In response, we have revised the opening sentences of the Introduction to focus on benthic ecosystem functioning rather than benthic remineralization, and aligned the opening sentences of the abstract accordingly to better link benthic ecosystem functioning with contrasting sea-ice regimes and their effects on organic matter supply and nutrient cycling.

**Comment:** L31 you incubated sediment cores in different location (in situ?) or you incubated cores collected from different locations (ex situ?)?

**Response:** Cores were collected from different locations and incubated on the ship, therefore ex situ. We have clarified this (page 2, line 42).

**Comment:** L33 If you measured oxygen and nutrients, maybe you could emphasize nutrient cycling in your opening sentence and not also carbon. Sure, some aspects of dissolved carbon fluxes are linked to consumption of oxygen, but you have many remineralization processes that do not use O2 as terminal electron acceptor. So you rather study oxygen consumption and not carbon cycling.

**Response:** In response, we have revised the opening of the abstract (and introduction) to focus more broadly on benthic ecosystem functioning rather than carbon cycling.

**Comment**: L49 Same as in the abstract. I don’t think carbon storage and cycling is really the topic you are studying.

**Response:** We have revised this section of the introduction to remove the emphasis on carbon storage and instead highlight the importance of seafloor habitats for benthic ecosystem functioning and sediment biogeochemical processes, which more accurately reflects the scope and objectives of our study (page 4, lines 89-98).

**Comment:** L50-52 This is imprecise and not correct as stated. Benthic communities don’t directly remove CO₂ from the atmosphere. They process organic carbon delivered from the water column. Also, “removing carbon” implies sequestration or burial. Recycling is quite the opposite of removal.

**Response:** We thank the reviewer for this important comment. Because carbon sequestration is not the primary focus of this study, we have removed this sentence from the introduction (see comment above).

**Comment:** L58-59 “Yet, the drivers of benthic remineralization are still poorly understood on regional scales.” I think this is not correct. Sure there is much that we do not know, but you do not provide any information about the drivers of remineralization. At least some mentioning of the redoxcline and the principles of the biogeochemical processes involved here should be mentioned. Organic matter quality, temperature, grainsize/porosity, bioturbation & bioirrigation. There is much we do know.

**Response:** Our intention was not to suggest that these fundamental drivers are unknown, but rather that their relative importance and interactions remain insufficiently understood in Arctic environments experiencing contrasting sea-ice regimes. Accordingly, we have revised the introduction to better reflect the current state of knowledge (page 4-5, lines 89–109).

**Comment**: L60 benthic biodiversity? Also substrate type is very important for benthic communities. I am not sure depth itself is a particularly important driver itself. It’s rather a proxy of the other drivers that change with depth.

**Response:** We have revised this section of the introduction accordingly to better reflect the relationships of depth and food availability and other factors and provide a more accurate ecological framework (page 4-5, lines 89-119).

**Comment:** L64-65 I am not sure these studies are so recent anymore. They are all at least 10 years old.

**Response:** We have removed the term "recent" while retaining the relevant references (page 5, line 123).

**Comment:** L63-64 can you provide some references for such historic studies?

**Response:** As part of the revision of the introduction, we removed this sentence.

**Comment**: L63-69 Can you be a bit more precise here? Sure functional traits are mainly defined to describe ecosystem functions. But what traits and modalities are relevant for what processes. And which relate actually to your study objective (remineralization and fluxes)?

**Reponse:** We now explicitly refer to feeding mode, mobility, bioturbation potential, body size. In particular, we clarify how these traits can influence oxygen consumption and nutrient fluxes by modifying organic matter processing, sediment mixing, and solute exchange across the sediment–water interface. This revision better links the functional trait framework to the objectives of our study (page 5-6, lines 120-133).

**Comment:** L69 same as before. 2008 or 2010 are not really recently.

**Response**: We removed the term “recently”.

**Comment:** L70-71 Isn’t this contradicting your previous statement that drivers are poorly understood on a regional scale?

**Response:** We agree that the original wording was contradictory. In the revised introduction, we acknowledge that key controls, such as the supply of labile organic matter, sediment characteristics, and redox conditions, are well established. We now clarify that the remaining knowledge gap concerns how these environmental and biological factors interact to regulate spatial variability in benthic ecosystem functioning under contrasting Arctic sea-ice regimes (page 5, lines 116-119).

**Comment:** L89-92 I don’t think your introduction really fits to your objectives. First, you hardly mention nutrients in your introduction. Why are they actually important and why would you want to study them. Second, why are you studying oxygen uptake? You haven’t mentioned oxygen so far. Third, you describe different ice conditions in the different subregion, but you never establish that they might be associated with different production and OM export regimes. Fourth, evaluating potential environmental drivers is fine, but you haven’t established based on literature what environmental drivers are important in other studies. Fifth, it’s not one process, it’s processes. It would be useful to also describe the different nutrients you consider and key components of their cycling. Besides, you do not study the fluxes of the benthic communities, you study the fluxes across the sediment water interface.

**Response:** We have substantially revised and reorganized the introduction to improve alignment of introduction and objectives

Specifically, we now (i) frame the study in terms of benthic ecosystem functioning rather than carbon cycling/remineralization, (ii) introduce the importance of oxygen and nutrient cycling, together with the sediment biogeochemical processes that regulate oxygen uptake and nutrient fluxes across the sediment–water interface, (iii) explain how contrasting sea-ice regimes influence primary production, organic matter export to the seafloor, and consequently benthic ecosystem functioning, (iv) better describe the environmental factors known to influence these processes, including organic matter supply, sediment characteristics, and redox conditions, and (v) revise the terminology throughout the manuscript to consistently refer to fluxes across the sediment–water interface rather than benthic fluxes.

**Comment:** L95-113 The study site description should be improved. One aspect is the language which should be improved, but also the content. Please adjust this to your study objective. You do not mention anything about benthic habitats, OM export, or productivity. Maybe bottom currents?

**Response:** We have revised this section to improve both the language and the scientific context. In particular, we now describe the contrasting sea-ice regimes, their influence on primary production and organic matter export to the seafloor, and sediment dynamics in the region. We also expanded the description of the hydrographic setting, including the role of bottom water circulation(page 9-10, line 199-228).

**Comment:** Table 1 Didn’t you write before that it is about 500m deep? Some of your stations are much deeper. Also you are inconsistent in using “North Water Polynya” and “Northern Baffin Bay”. This should be at least clarified. I am not sure that longitude should be reported with – if you also write that it is degree W.

**Response:** We have removed this sentence to avoid the apparent discrepancy between the general bathymetric description and the depths of the sampling stations (Page 9, line 204-208). We also standardized the terminology throughout the manuscript by using “northern Baffin Bay” instead of “North Water Polynya” when appropriate. Finally, we removed the longitude in Table 1.

**Comment:** L128 it’s not nutrient remineralization… you look at nutrient fluxes. You cannot know if remineralization is ongoing during your experiments or if just the dissolved nutrients in the porewater are released.

**Response:** We have revised the terminology throughout the manuscript to refer to oxygen and nutrient fluxes across the sediment–water interface.

**Comment:** L128-131 you did all of this with one small syringe sample? I guess not, so please correct the text. Besides, I find the syringe size and sampling depth of 1 cm appropriate for pigment samples. But for OM content and grain size, I think larger volumes and deeper penetration depth should be used. Also, if you did 1 replicate, what are the numbers between the +- sign showing?

**Response:** We have revised the methods to describe the different sediment sampling procedures in more detail (Page 10-14, line 236-266). Specifically, three small syringe cores (0–1 cm) were collected from each box core for the determination of chlorophyll *a* and phaeopigments. In contrast, only one larger sediment core (60 mL syringe) was collected from each box core for grain-size (n=1) and organic matter analyses (n=1) in order to minimize the amount of sediment removed from the box core.

For grain-size analysis, the upper 5 cm of sediment from the large core were used for laser granulometry. For organic matter content, only the upper 1 cm was analyzed by loss-on-ignition because this surface layer is the most biogeochemically active, directly participates in exchanges across the sediment–water interface, and contains the most recently deposited organic matter. Consequently, it is the sediment layer most relevant to the objectives of the present study.

**Comment:** L134-137 maybe field sampling is the wrong section for this?

**Response:** We have moved these sentences to a small section on “Sea ice cover” (Page 21, line 424).

**Comment:** L139-140 I find this very limited for actually describing your core variables you are trying to explain. You measure all these variables, but they all are linked to different processes: e.g. respiration, nitrogen cycle processes, phosphorus regeneration or dissolution of biogenic Silica. Not all of these fluxes are necessarily indicating OM remineralization. Phosphate can be released from iron bound P. Dissolved Silica comes primarily from dissolution of biogenic silica. For example diatom frustules. These frustules are not OM themselves.

**Response:** Thank you for this important comment. To better reflect the scope of our measurements, we have replaced the term "benthic remineralization" with "benthic ecosystem functioning" throughout the manuscript. We also revised the Methods to clarify that the measured oxygen and nutrient fluxes represent complementary indicators of sediment biogeochemical functioning rather than direct measurements of remineralization alone (page 14-15, line 268-307).

**Comment:** L143-144 Are these the same cores you mentioned in L126-8? Or are they different?

**Response:** These are the same cores mentioned in L138-140.

**Comment:** L144 I understand that you had probably limited possibilities to reduce the temperature below 4 degree, but according to table 1, you study sites have bottom temperatures were between -0.2 and 1.5 degrees. Considering the great depth, the fauna and microbes in your cores are very unlikely to ever experience such high temperatures in their natural environment. This is a very important limitation of your experiment and needs to be considered carefully when interpreting your results.

**Response:** Indeed, we were not allowed to reduce the temperature of the cold-room to below 4 degrees to prevent any problems with the cooling systems. We now clearly acknowledge that the difference to in situ temperatures may have increased the metabolic activity of both benthic fauna and microorganisms and therefore influenced the absolute magnitude of the measured oxygen and nutrient fluxes. Because all cores were incubated under identical temperature conditions, we expect that the relative differences among stations remain meaningful for evaluating spatial patterns in sediment–water interface fluxes. We have now explicitly discussed this limitation in the discussion and emphasize that our results should be interpreted primarily in terms of relative comparisons among stations rather than absolute in situ flux rates (page 40, lines 846-851).

**Comment**: L146 is it really for settling of particles or rather for the fauna to behave more normally after the stress of being collected.

**Response**: The purpose of this acclimation period was not only to allow suspended particles to settle but also to minimize the effects of sampling disturbance by allowing the sediment cores and benthic fauna to stabilize before the incubations began. We have revised the methods accordingly to clarify the rationale for the 6–8 h acclimation period (page 14, line 270-292).

**Comment:** L147-151 I understand that this is pretty standard procedure for these experiments, but according to table 1 the DO you measured in-situ was not so high. At your temperature the 240uM that you have on average at your stations is probably around 70% saturation. So by oxygenating your cores to 100%, you add oxygen in comparison to in-situ. And then you even stop your experiment at 80% saying that below this the biogeochemistry might change. But if in-situ oxygen levels are lower than this, then your argument would suggest that your experiment was conducted under conditions that do not reflect the in-situ biogeochemical processes.

**Response:** This procedure was adopted to provide standardized initial conditions for all incubations and is commonly used in benthic core incubation experiments to avoid oxygen limitation during the measurements (provide 2 refs). However, we acknowledge that these conditions may not fully reproduce the natural bottom-water oxygen concentrations and may therefore have influenced the absolute magnitude of the measured oxygen and nutrient fluxes.

Our rationale for terminating the incubations at approximately 80% air saturation was to avoid substantial oxygen depletion during the experiment, again following the common protocol. We have revised the methods (page 15, lines 298-301) and expanded the discussion (page 40, lines 843-857) to clarify this distinction and explicitly acknowledge that, although the absolute fluxes should be interpreted with caution, the standardized incubation protocol allows robust comparisons of relative spatial differences among stations because all sediment cores were subjected to identical experimental conditions.

**Comment:** L156-158 You are not measuring oxygen flux, you measure oxygen concentration and then you calculate the flux from the change in concentration. Did you really measure continuously? Or rather frequently? I assume you calibrated the optode before the cruise?

**Response:** “Oxygen flux” was changed to “oxygen concentration” (page 16, line 310-314). The oxygen concentration was measured continuously with a Fibox 4 (<https://www.presens.de/products/detail/fibox-4>) using oxygen sensor spots (<https://www.presens.de/products/detail/oxygen-sensor-spot-sp-pst3-yau>) directly attached to the incubation cores. The optodes were calibrated before the cruise according to the manufacturer’s recommendations: <https://www.presens.de/support-services/faqs/question/how-can-i-prepare-the-calibration-solutions-cal0-and-cal100-for-oxygen-sensors-35>

**Comment:** L159-160 how did you deal with replicate cores consuming O2 at different rates? Did you still collect samples from every core of the experiment at the same time? OR did you do this individually for each core? And what about your control cores with only bottom water?

**Response:** Each core was treated individually, including the controls, meaning that water samples for nutrient analyses were not necessarily collected at the same time between the treatments. Although there were differences in oxygen consumption between the different cores, these differences were only a matter of a few hours at most.

**Comment:** L161-162 what do you mean with known solute concentration? Did you collect a sample of the bottom water for nutrient analysis everytime you replaced some water in your cores?

**Response:** Yes, a sample of bottom water was collected and analyzed every time we replaced water in the cores. These measured concentrations were then used to correct the nutrient flux calculations for the nutrients introduced with the replacement water, based on the exact volume exchanged. We have clarified the methods to point out this ambiguity (page 16, lines 318-322).

**Comment:** L164 Have you tested whether using GF/F filters actually affects your dissolved silica concentrations?

**Response:** We did not specifically test the potential effect of Whatman GF/F filtration on dissolved silica concentrations. All samples were filtered using the same protocol and the same type of filter. Prior to filtration, GF/F filters were rinsed with sample water, further minimizing any potential contamination. However, we acknowledge that there are growing concerns in the scientific community about the use of such filters because they are composed of borosilicate glass, which could potentially introduce a small systematic bias in dissolved silicate measurement.

**Comment:** L166 So ammonium was measured from a different sample than the other nutrients?

**Response**: Yes, ammonium was measured from an additional water sample because it was analyzed on-board using a fluorimeter. We have clarified the methods (page 16, line 322).

**Comment:** L165-169 please provide some information on the used standards and measurement error.

**Response:** Information were added in the methods (page 17, line 331).

**Comment:** L170-172 You also need to correct for the water volume in your core. You wrote the sediment cores were 15-20cm long. This makes quite a difference and needs to be corrected for after you corrected for your control cores.

**Response:** Every cores were individually measured (internal diameter, sediment depth, water level) at the end of the incubations in order to calculate the exact amount of water in the cores. We have clarified this ambiguity (page 17, line 332-338).

**Comment:** L183 what base for the logarithm?

**Response**: The index was calculated using the natural logarithm (ln). We have specified it in the methods (page 17, line 346-347).

**Comment:** L190 I agree in general with the traits you selected, but you should justify why you consider each of them relevant. Maybe already in the introduction.

**Response:** We have therefore expanded the introduction to explain why these traits were considered relevant to sediment–water interface oxygen and nutrient fluxes (page 5, line 120-133).

**Comment:** L235 why just the top centimeter? How does this relate to the depth were your remineralization processes are happening and your fauna is active?

**Response:** As stated above regarding your comment about L128-131, we selected the upper 1 cm of sediment because it is the sediment layer most relevant to the objectives of the present study. It represents the sediment layer most directly involved in exchanges across the sediment–water interface. It is also the most biogeochemically active which directly participates in exchanges across the sediment–water interface and contains the most recently deposited organic matter.

**Comment:** L243-272 please be more precise in how you describe your data analysis. I am not completely able to follow and would not be able to repeat your analysis.

**Response:** We have revised the data analysis section to describe each analytical step more explicitly, including the data transformations applied, the construction of the environmental, taxonomic, and functional datasets, the criteria used for variable selection, the statistical methods employed, and the software packages and settings used. We believe these revisions improve the clarity and reproducibility of the analyses (page 21-23, line 430-485).

**Comment:** L246 and what does the ‘missing’ function do?

**Response:** The PRIMER *missing* function was used to estimate missing values using the Expectation–Maximization (EM) algorithm, which exploits the correlation structure among variables to obtain maximum-likelihood estimates for incomplete observations. This procedure allowed us to complete the data matrix prior to the multivariate analyses. We have clarified this in the Methods (page 21, lines 435-437).

**Comment:** L248 appropriate pair-wise comparison?

**Response:** We have revised the methods to remove the term “appropriate” (page 21, line 438).

**Comment**: L253-256 What are these different subsets that you used in the separate analyses?

**Response:** By "subsets," we were referring to the three groups of explanatory variables that were analyzed separately: (i) environmental variables, (ii) taxonomic diversity indices, and (iii) functional trait indices that are presented in the methods just before. We have revised the methods to explicitly define these subsets and describe the variables included in each model (page 21, lines 442-474).

**Comment**: L259 you need to also provide information on what variables they correlated with. Otherwise it is impossible to interpret your results correctly. You are making an arbitrary choice in what you exclude.

**Response**: We have revised the methods to explicitly report the pairs of variables that exceeded the correlation threshold, identify which variables were retained for subsequent analyses, and explain the rationale for these decisions based on ecological relevance and statistical redundancy (page 22, lines 450-469).

**Comment:** L275 These general pattern suggests that you have probably altered redox conditions in the sediment through the oxygenation of the water in your cores. In particular that NH4+ was taken up by the sediments across all your experiments and that in parallel NO3- was released in large amounts is almost certainly partly caused by your sample treatment. I will explain. As I mentioned above, you started with not fully saturated bottom water (in-situ). Your oxygenation of the cores increases the saturation (typically close to 100%) and also reduces the diffusive boundary layer. Consequently, oxygen is penetrating deeper into your sediments, which activates nitrifiers. So the pattern you see is indicating that you artificially increased nitrification (i.e. NH4+ is consumed and NO3- produced). This is not completely invalidating your experiments, but you need to consider this carefully when interpreting your data.

Also, this highlights a key problem in the story you are telling. It’s not all remineralization! Fluxes also reflect other biogeochemical processes, like nitrogen transformation.

**Response:** We agree that oxygenating the overlying water before the incubations likely increased oxygen penetration into the surface sediments relative to in situ conditions, potentially stimulating aerobic microbial processes such as nitrification. This mechanism provides a plausible explanation for the concurrent uptake of NH₄⁺ and release of NO₃⁻ observed in several incubations. We have expanded the discussion to explicitly acknowledge that the incubation procedure may have altered the sediment redox environment and influenced the absolute magnitude of the measured oxygen and nutrient fluxes (page 38-41, lines 811-863).

However, because all sediment cores were subjected to the same experimental protocol, we consider that the relative differences among stations remain appropriate for assessing the influence of environmental gradients and benthic community characteristics. We have also revised the manuscript to avoid interpreting the measured fluxes solely in terms of remineralization. Instead, we now emphasize that sediment–water interface oxygen and nutrient fluxes integrate multiple sediment biogeochemical processes, including organic matter mineralization, nitrification, denitrification, and other nitrogen transformations, which are influenced by sediment redox conditions.

**Comment:** L276 Did you introduce this abbreviation? What does NOW stand for?

**Response:** We have revised the manuscript to define NOW as North Water Polynya at its first occurrence but, as stated before, we also standardized the terminology to keep “northern Baffin Bay” instead of “North Water Polynya” when possible. Northern Baffin Bay is the correct geographical location, while “NOW” refers to an ecological defined area.

**Comment:** L277&281 I don’t understand why you present oxygen in mmol and SiO2 in micromole. They span almost exactly the same range of values, but one is now given as max 4.4 mmol and the other one as max 4800 micromol.

**Response:** To improve consistency and readability, we have revised the manuscript to report silicic acid fluxes in mmol m⁻² d⁻¹, consistent with the oxygen fluxes, while retaining the original units for other fluxes (Fig 3)

**Comment:** L279-282 That’s not correct, your figure 2 does not show the same pattern for O2, PO43-, DSi, and NH4+.

**Response:** We agree that the original wording implied that O₂, PO₄³⁻, Si(OH)_4_, and NH₄⁺ exhibited the same spatial pattern, which is not supported by Figure 2. We have revised the text to describe the spatial distribution of each flux more accurately and to avoid suggesting a common pattern where differences exist (page 25, lines 516-531).

**Comment:** L281 It’s actually more appropriate to report the Silica as DSi and not SiO2. The molybdate blue method measures dissolved, reactive silica (DSi) and this is also the form in which the fluxes occur. Writing SiO2 is giving the impression that your fluxes would be in solid mineral form.

**Response:** To avoid ambiguity and follow current terminology in marine biogeochemistry, we have revised the manuscript to refer to these measurements as silicic acid (Si(OH)_4_) throughout the text, tables, and figures. Si(OH)_4_ was chosen over DSi to align with the papers cited in the references.

**Comment:** L283-286 This indicates that at RA67 you had incomplete nitrification.

**Response:** Thank you for this very interesting remark. As stated above, we have revised the discussion to acknowledge the limits of the incubations as well as spatial differences in nitrogen transformations and explain potential reasons for such differences (page 38-41, lines 811-863).

**Comment:** L292-3 This statement is not correct. PERMANOVA tests for differences in group means. It’s not testing variability.

**Response:** We have revised the text accordingly to state that PERMANOVA identified significant differences in benthic fluxes among stations. Differences in multivariate dispersion were evaluated separately using PERMDISP and are reported independently (page 26, line 551-571).

**Comment**: L294 PERMDISP is addressing variability, but only to test whether dispersion (variability) differed between the different groups. Not comparing within vs among experiment variation. Your previous statement is still not accurate according to your tests, but in general the PERMDISP supports that permanova result is not related to differences in dispersion. Anyhow, please keep in mind that your PERMDISP has very low power due to the low number of replication in each experiment (n=3).

**Response**:. We have revised the text to state that PERMANOVA detected significant differences in multivariate benthic fluxes among stations, while PERMDISP indicated no significant differences in multivariate dispersion among stations. We also acknowledge that, because each station was represented by only three replicate cores, the PERMDISP test had limited statistical power, and its results should therefore be interpreted with caution (page 26, lines 551-557).

**Comment**: L296 For the pairwise comparisons you should apply a p-value correction for multiple testing. With alpha = 0.05, you have to expect that on average you have 2 false positives for the 36 pairwise comparisons you are running to compare all your experiments.

**Response**: We have therefore applied the Holm–Bonferroni correction to the pairwise PERMANOVA *p*-values and revised the results accordingly. The corrected *p*-values are now reported in the manuscript (page 27, line 560-553).

**Comment**: L298-299 NMDS is a visualization of a dismilarity matrix. It does not test for differences in across and within site variability. It’s correct that station RA67 is very different from all the other. You could make this already clear when you describe Figure 2. Maybe for the nMDS you could consider excluding this station. It seems like this difference is so dominant that you might miss patterns in the remainder of your experiment.

**Response:** We have therefore revised the text to avoid attributing statistical inference to the NMDS and instead describe the observed ordination patterns. We also agree that station RA67 is clearly separated from the other stations and have already highlighted this observation when describing the results. Following your suggestion, we evaluated the effect of excluding RA67 from the ordination to improve visualization of the remaining stations. However, because RA67 represents a genuine sampling station rather than an outlier, we retained it in the main ordination to provide a complete representation of the dataset (page 28, line 582, Fig 4).

**Comment**: L331-334 I don’t really understand why you are not also considering the environmental variables as another set in the variation partitioning. And similarly, why you did not separate functional from taxonomic diversity in the distLM before.

**Response:**Environmental variables were indeed included as a separate explanatory set in the variation partitioning analysis, together with taxonomic diversity and functional diversity (page 23, line 463-463 in the methods)(page 30, line 623-638, Fig 6 in the results).

For the DistLM analyses, taxonomic and functional diversity variables were intentionally considered together as a single "diversity" predictor set. Our objective at this stage was to contrast the overall contribution of biological diversity with that of environmental variables, whereas the subsequent variation partitioning analysis was specifically designed to disentangle the relative contributions of environmental, taxonomic, and functional components. We have clarified this rationale in the manuscript (page 21, lines 446-450).

**Comment:** Besides, I don’t follow the description in the methods completely, but it sounded like you had a very small set of taxonomic diversity measures that you considered (Shanon, Pielou, taxon richness). But for functional diversity you use the three diversity measures and the CWM for all 16 modalities. So you consider 19 functional variables… This is very unbalanced and it’s obvious that this is largely causing your difference between functional and taxonomic diversity.

**Response:** We agree that the functional predictor set initially contained more candidate variables than the taxonomic diversity set and that a larger initial pool of candidate variables may increase the opportunity to identify significant predictors. However, the interpretation of our results is based on the final DistLM models rather than the initial candidate sets. Only three functional variables were retained following model selection, while the remaining functional variables did not contribute to the final model. Model selection was based on adjusted R², which penalized model complexity.

**Comment:** Overall, what I missed completely from the result section is a description of the benthic communities (taxonomic and functional). After reading your paper I do not have any idea what benthic communities were in your cores.

**Response**: Indeed, this was one of the main limitation of the current version of the paper. We have now added a description of the communities in the results (page 23, line 475-489), a graph (Fig 2, page 25, line 514) about the functional traits found at each stations as well as abundance data in Table 1 (page 13, line 260).

**Comment:** In general, the discussion lacks any consideration of biogeochemistry, which is extremely limiting. Also, for much of the discussion it is not really clear to me what the authors want to achieve with what they wrote. Much of what appears here should have probably been parted of the introduction and not written in the discussion where it is only loosely related to your results. Since the issues pointed out above are expected to change the results, I am providing less detailed comments on the discussion.

**Response:** We have completely revised the discussion to focus more directly on the interpretation of our results in the context of sediment biogeochemistry, including the roles of oxygen availability, organic matter degradation, redox conditions, and nutrient cycling. We have also reorganized the discussion so that each section is structured around the main findings of the study, while moving general background information to the Introduction. We believe these revisions have improved the focus and interpretation of our results.

**Reviewer #2
Results**

**Comment:** The authors report that the proportion of deposit-feeders is an important driver of fluxes; however, the data on the community structure at these sites is only contained in supplemental tables, with little granularity as weighted means. It would be helpful to show at least the proportion of the different functional groups among the different stations in the main text as a figure so the reader could get a sense of how communities vary among the sites, which in itself provides a lot of info on what the environment is like.

**Response:** We have added a description of the communities in the results (page 23, line 487-501), a new figure (Fig. 2) showing the main functional groups found at each station and data about abundance/biomass in Table 1 (page 13).

**Comment:** There is no abundance or biomass information presented in the results to give a sense of how distribution of biomass among taxa affected the results, even though CWMs turned out to be significant in the analysis. For example, are deposit feeders important because they are deposit feeders, or is it just that they were the most abundant (or largest) organisms and therefore had an outsized effect on fluxes? How did average body size vary (or not) among sites? This consideration of the differences in communities across the sites should be better incorporated into the discussion of why fluxes vary among sites, especially given that the stated aim of the paper is to consider functional diversity as a control on fluxes. There is literature on how specific species or types of deposit feeders affect nutrient fluxes as well (including direction of flux), which is not considered here.

**Response:** We have expanded the results to better describe the distribution of the benthic communities among stations, including the relative abundance/biomass in Table 1 (page 13) and body-size composition in Figure 2 (page 25, line 514). We have also revised the discussion to better relate the observed spatial variation in community composition to the significant community-weighted mean (CWM) traits identified by the DistLM analysis.

We acknowledge that CWM traits are influenced by the relative abundance of taxa and therefore do not distinguish whether the observed relationships arise from the functional traits themselves or from the dominance of particular taxa possessing those traits. Accordingly, we have revised the discussion to interpret these relationships as associations rather than direct causal mechanisms.

**Comment:** There are two functional types of deposit feeders listed in table 2, but only “deposit feeders” appears in the analysis. Are they lumped together for the stats?

**Response:** The two deposit-feeding modalities were intentionally combined into a single deposit-feeder category prior to the analyses. Our objective was to represent deposit feeding as a broad functional strategy because both modalities contribute to the processing of sediment organic matter and sediment reworking, despite differences in their feeding depth. The two modalities were combined to reduce the number of highly related predictor variables and avoid unnecessary model complexity (point raised by reviewer #1). We have clarified this in the table 2 (page 18, line 372).

**Comment:** How does the size of deposit feeders matter impact the results? Because “CWM of small organisms” is also identified as important but there is no explanation of what that means exactly.

**Response:** The community-weighted mean (CWM) of small organisms reflects communities dominated by small-bodied taxa rather than simply the presence of small individuals. We have expanded the discussion to explain how body size may influence sediment biogeochemical processes through differences in metabolic activity, sediment reworking, and bioirrigation, while acknowledging that our analyses identify associations rather than direct causal relationships. We have also clarified that body size may act as an indicator of broader differences in benthic community composition and ecosystem functioning among stations (page 37, line 766-782).

**Comment:** The Results section is organized around the statistical methods used, and not around the questions asked. I suggest reorganizing based on the stated objectives at the beginning. For example, the two sections on “spatial variation” could be combined, and the “variance partitioning” section doesn’t need to stand alone. A suggested organization might be 1) Variation in flux among sites, and 2) Drivers of flux (be that environmental or faunal driven). It would make the presentation of results more impactful and clearer.

**Response:** We have substantially reorganized the Results section as you suggested. The previous sections describing spatial variation have been combined into a single section presenting the variation in benthic fluxes among stations, while the results from the DistLM and variation partitioning analyses are now integrated into a section describing the environmental and biological drivers of benthic fluxes. We have also added a section about the taxonomic and functional structure of the benthic communities. We believe this revised structure better reflects the objectives of the study and makes the presentation of the results clearer and more impactful (page 23-32, line 476-660).

**Comment:** Discussion - I personally like to see a brief summary statement at the start of the discussion that highlights key results, which makes for easy reference and indicates what the authors thought were the most important take-away messages as the results section can somethings obscure the key messages with all the technical details.

**Response:** We have added an opening paragraph summarizing the key results and the main take-home messages of the study (page 31, line 662-682).

**Comment:** - The first section of the results (beginning line 341) goes into some detail comparing the values measured here to values measured elsewhere. While this is important information to include, what is the actual point being made in this paragraph with respect to the objectives of the project? There is no topic sentence for this section and it reads like an annotated bibliography.

**Response**: We have removed this paragraph to improve the clarity and focus of the section.

**Comment:** - Line 378: “explain the patterns found” What patterns? First, describe the patterns you are referring to. I agree that you need to consider the potential role of faunal components not measured, but this passing reference to micro and meiofauna doesn’t really do that point justice. This could be expanded upon a bit to better consider their role.

**Response:** We agree that the phrase "patterns found" was imprecise and have replaced it with "unexplained variation" to more accurately reflect the results of our analyses. We have also added a section in the Discussion to consider the potential sources of the unexplained variation in benthic fluxes, including the possible influence of unmeasured biological components such as meiofauna and microbial communities, as well as other environmental factors not included in our models (page 38, line 800).

**Comment:** - A new section on environmental drivers is not needed. In the “Variability” section, you should more explicitly describe the variability you refer to, and then follow up with an explanation of the possible reasons for that variability (environmental or biological drivers)—these aren’t separate ideas that merit separate subheaders.

**Response:**	We have removed the separate section on flux variability and substantially reorganized the Discussion also based on reviewer’s #1 comments. The revised structure now includes two complementary sections focusing on (i) the role of hydrography in shaping sediment characteristics and associated redox conditions and (ii) the effects of benthic community composition on flux variation (page 31-40, line 662-853). This reorganization provides a clearer progression from the observed patterns to the environmental and biological mechanisms that likely explain them.

**Comment:** - Section beginning line 394. Seems to suggest sea ice is important because it mediates grain size? But doesn’t it also mediate organic matter flux, which would seem quite important to remineralization rate AND community structure?

**Response:** We have revised the Discussion to emphasize that sea-ice conditions likely influence oxygen and nutrient fluxes through multiple interacting pathways, including the regulation of primary production and the quantity and quality of organic matter reaching the seafloor, in addition to their indirect influence on sediment characteristics through hydrographic processes. We also discuss how these mechanisms may affect sediment redox conditions and benthic community composition, thereby providing a more comprehensive interpretation of the observed environmental controls on benthic fluxes (page 33, line 687-714).

**Comment**: - I also wonder if the effect of sea ice in the analysis is driven entirely by the one station nearest the ice, which seems to sit apart from everything else in the ordination plots.

**Response:** We agree that it is important to consider whether the observed relationship could be disproportionately influenced by a single station. However, the sea-ice gradient in our study was not represented by only one station. At the time of sampling, two stations (RA38 and the northernmost station RA25) were not ice-free, indicating that the effect of sea ice was not solely associated with a single observation (Table 1, page 13).

**Comment:** - Last few paragraphs beginning line 442: In my opinion, this is the most interesting part of the study, but there are factually incorrect statements and tenuous assertions made. In some cases, the references are not appropriate for some of the statements made.

**Response:**. Upon reconsideration, we concluded that this section extended beyond the scope of the present work. While it may help explain patterns in benthic community composition, it was not directly supported by our results on benthic oxygen and nutrient fluxes. Rather than substantially revising these interpretations, we chose to remove this section from the Discussion to maintain a clearer focus on the environmental and biological drivers of benthic fluxes identified by our analyses. Consequently, the subsequent comments pertaining specifically to this section are no longer applicable.

**Comment:** - There is no consideration in this section of which types of deposit feeders were found in this study at these sites, yet there is reference to specific modes (e.g. lines 467 – 469; 473 - 475) found to be important in other areas. What is the link to the data from this study? Are these modes found in this dataset? Explore this idea further, and again bring in the specific results about the community to the results section.

**Response:** stated previously, in the functional trait analysis, the two deposit-feeding modalities were intentionally combined into a single deposit-feeder category because our objective was to represent deposit feeding as a broad functional strategy rather than distinguish between finer-scale feeding modes. We agree that the previous version of the Discussion placed too much emphasis on mechanisms associated with specific types of deposit feeders described in the literature without adequately linking them to our own dataset. We have therefore substantially revised this section. Rather than discussing the effects of particular deposit-feeding modes, the revised Discussion focuses on the broader ecological interpretation supported by our results. Specifically, we discuss how the observed relationship between deposit feeders and benthic fluxes likely reflects their shared response to the same environmental drivers—namely sea-ice cover, sediment grain size, and water depth—which are known to regulate both benthic fluxes and the distribution of deposit-feeding communities in Arctic sediments. We believe this revised interpretation is more consistent with the level of resolution of our functional trait analysis and is better supported by our data.

**Comment:** Line 490 refers to “two distinct regions” that were identified. I missed where this was presented in the results?

**Response:** We have revised the Discussion to remove this wording and to focus on the observed environmental gradients and spatial variation directly supported by our analyses.

**Comment:** - Line 497: Our findings provide crucial support… Explain further, identifying clear links between these data and “assessing the ability of benthos to process carbon in response to climate change”. These results were not presented in the context of climate change per se.

**Response:**. We have completely revised the Conclusion to focus primarily on the main findings of the present study before placing them in a broader Arctic context. Rather than making direct inferences about climate change impacts, the revised text highlights how the environmental gradients investigated in Nares Strait provide a useful framework for understanding how changes in sea ice and hydrographic conditions may influence benthic ecosystem functioning in similar Arctic marine systems. We believe this revised conclusion more accurately reflects the scope of our data and the implications supported by our results (page 41).
