# Supplementary material for: Hydrographic and sea ice gradients shape benthic ecosystem functioning through sediment characteristics and benthic functional structure in Nares Strait, Canadian Arctic
Source: PLoS One. 2026 Nov 13;21(11):e0345559. doi: 10.1371/journal.pone.0345559 (PMC13766610; doi:10.1371/journal.pone.0345559)
Supplement: Peer review history

# Peer review: Decision Letter 0

**DOI:** 10.1371/journal.pone.0345559.r001

**Authors:** Clara Rodrigues

30 Apr 2026

Dear Dr. Combaz,

Thank you for submitting your manuscript to PLOS ONE. After careful consideration, we feel that it has merit but does not fully meet PLOS ONE’s publication criteria as it currently stands. Therefore, we invite you to submit a revised version of the manuscript that addresses the points raised during the review process.

As you will see, both reviewers find your study on benthic fluxes and faunal influence in the Arctic to be timely and your data highly valuable. However, both have raised substantial concerns that must be addressed before the paper can be considered for publication.

The primary issues involve a lack of integration between your faunal data and biogeochemical processes, as well as significant structural weaknesses in the presentation of your results and discussion. Specifically, I would like you to focus on the following in your revision:

Biogeochemical Integration: Reviewer 1 notes that the manuscript attempts to study faunal influence on biogeochemistry without sufficient consideration of the underlying biogeochemical processes. This gap affects the interpretation of your results and must be addressed throughout the text.

Methodological Transparency: While your results are not considered invalid, several methodological shortcuts need to be discussed openly to ensure the reader can properly contextualize your findings.

Structural Reorganization: The Results section should be reorganized around your research questions/objectives rather than your statistical tests. Additionally, the Discussion requires a thorough editorial polish to improve flow, ensure every paragraph has a clear topic sentence, and remove redundant phrasing.

Please move key community structure data (abundance, biomass, and functional groups) from the supplements into the main text. The reviewers need to see why certain groups, like deposit feeders, are driving fluxes—whether it is due to their specific functional traits or simply their biomass.

Please provide a point-by-point response to the detailed comments attached below.

Please submit your revised manuscript by Jun 14 2026 11:59PM. If you will need more time than this to complete your revisions, please reply to this message or contact the journal office at plosone@plos.org. When you're ready to submit your revision, log on to https://www.editorialmanager.com/pone/ and select the 'Submissions Needing Revision' folder to locate your manuscript file.

- A letter that responds to each point raised by the academic editor and reviewer(s). You should upload this letter as a separate file labeled 'Response to Reviewers'.
- A marked-up copy of your manuscript that highlights changes made to the original version. You should upload this as a separate file labeled 'Revised Manuscript with Track Changes'.
- An unmarked version of your revised paper without tracked changes. You should upload this as a separate file labeled 'Manuscript'.

If you would like to make changes to your financial disclosure, please include your updated statement in your cover letter. Guidelines for resubmitting your figure files are available below the reviewer comments at the end of this letter.

If applicable, we recommend that you deposit your laboratory protocols in protocols.io to enhance the reproducibility of your results. Protocols.io assigns your protocol its own identifier (DOI) so that it can be cited independently in the future. For instructions see: https://journals.plos.org/plosone/s/submission-guidelines#loc-laboratory-protocols. Additionally, PLOS ONE offers an option for publishing peer-reviewed Lab Protocol articles, which describe protocols hosted on protocols.io. Read more information on sharing protocols at https://plos.org/protocols?utm_medium=editorial-email&utm_source=authorletters&utm_campaign=protocols.

As the corresponding author, your ORCID iD is verified in the submission system and will appear in the published article. PLOS supports the use of ORCID, and we encourage all coauthors to register for an ORCID iD and use it as well. Please encourage your coauthors to verify their ORCID iD within the submission system before final acceptance, as unverified ORCID iDs will not appear in the published article. *Only*  the individual author can complete the verification step; PLOS staff *cannot*  verify ORCID iDs on behalf of authors.

We look forward to receiving your revised manuscript.

Kind regards,

Clara F. Rodrigues

Academic Editor

PLOS One

Journal Requirements:

When submitting your revision, we need you to address these additional requirements.

1. Please ensure that your manuscript meets PLOS ONE's style requirements, including those for file naming. The PLOS ONE style templates can be found at

https://journals.plos.org/plosone/s/file?id=wjVg/PLOSOne_formatting_sample_main_body.pdf and

https://journals.plos.org/plosone/s/file?id=ba62/PLOSOne_formatting_sample_title_authors_affiliations.pdf

2. In your Methods section, please provide additional information regarding the permits you obtained for the work. Please ensure you have included the full name of the authority that approved the field site access and, if no permits were required, a brief statement explaining why.

3. We note that the grant information you provided in the ‘Funding Information’ and ‘Financial Disclosure’ sections do not match.

When you resubmit, please ensure that you provide the correct grant numbers for the awards you received for your study in the ‘Funding Information’ section.

4. Thank you for uploading your study's underlying data set. Unfortunately, the repository you have noted in your Data Availability statement does not qualify as an acceptable data repository according to PLOS's standards.

At this time, please upload the minimal data set necessary to replicate your study's findings to a stable, public repository (such as figshare or Dryad) and provide us with the relevant URLs, DOIs, or accession numbers that may be used to access these data. For a list of recommended repositories and additional information on PLOS standards for data deposition, please see https://journals.plos.org/plosone/s/recommended-repositories.

5. Please include your full ethics statement in the ‘Methods’ section of your manuscript file. In your statement, please include the full name of the IRB or ethics committee who approved or waived your study, as well as whether or not you obtained informed written or verbal consent. If consent was waived for your study, please include this information in your statement as well.

6. We note that Figure 1 in your submission contain map images which may be copyrighted. All PLOS content is published under the Creative Commons Attribution License (CC BY 4.0), which means that the manuscript, images, and Supporting Information files will be freely available online, and any third party is permitted to access, download, copy, distribute, and use these materials in any way, even commercially, with proper attribution. For these reasons, we cannot publish previously copyrighted maps or satellite images created using proprietary data, such as Google software (Google Maps, Street View, and Earth). For more information, see our copyright guidelines: http://journals.plos.org/plosone/s/licenses-and-copyright.

We require you to either (1) present written permission from the copyright holder to publish these figures specifically under the CC BY 4.0 license, or (2) remove the figures from your submission:

1. You may seek permission from the original copyright holder of Figure(s) [#] to publish the content specifically under the CC BY 4.0 license.

We recommend that you contact the original copyright holder with the Content Permission Form (http://journals.plos.org/plosone/s/file?id=7c09/content-permission-form.pdf) and the following text:

“I request permission for the open-access journal PLOS ONE to publish XXX under the Creative Commons Attribution License (CCAL) CC BY 4.0 (http://creativecommons.org/licenses/by/4.0/). Please be aware that this license allows unrestricted use and distribution, even commercially, by third parties. Please reply and provide explicit written permission to publish XXX under a CC BY license and complete the attached form.”

Please upload the completed Content Permission Form or other proof of granted permissions as an "Other" file with your submission.

In the figure caption of the copyrighted figure, please include the following text: “Reprinted from [ref] under a CC BY license, with permission from [name of publisher], original copyright [original copyright year].”

2. If you are unable to obtain permission from the original copyright holder to publish these figures under the CC BY 4.0 license or if the copyright holder’s requirements are incompatible with the CC BY 4.0 license, please either i) remove the figure or ii) supply a replacement figure that complies with the CC BY 4.0 license. Please check copyright information on all replacement figures and update the figure caption with source information. If applicable, please specify in the figure caption text when a figure is similar but not identical to the original image and is therefore for illustrative purposes only.

The following resources for replacing copyrighted map figures may be helpful:

USGS National Map Viewer (public domain): http://viewer.nationalmap.gov/viewer/

The Gateway to Astronaut Photography of Earth (public domain): http://eol.jsc.nasa.gov/sseop/clickmap/

Maps at the CIA (public domain): https://www.cia.gov/library/publications/the-world-factbook/index.html and https://www.cia.gov/library/publications/cia-maps-publications/index.html

NASA Earth Observatory (public domain): http://earthobservatory.nasa.gov/

Landsat: http://landsat.visibleearth.nasa.gov/

USGS EROS (Earth Resources Observatory and Science (EROS) Center) (public domain): http://eros.usgs.gov/#

Natural Earth (public domain): http://www.naturalearthdata.com/

7. If the reviewer comments include a recommendation to cite specific previously published works, please review and evaluate these publications to determine whether they are relevant and should be cited. There is no requirement to cite these works unless the editor has indicated otherwise.

[Note: HTML markup is below. Please do not edit.]

Reviewers' comments:

Reviewer's Responses to Questions

**Comments to the Author**

1. Is the manuscript technically sound, and do the data support the conclusions?

Reviewer #1: Partly

Reviewer #2: Partly

**********

2. Has the statistical analysis been performed appropriately and rigorously?

Reviewer #1: I Don't Know

Reviewer #2: Yes

**********

3. Have the authors made all data underlying the findings in their manuscript fully available?

The PLOS Data policy

Reviewer #1: No

Reviewer #2: Yes

**********

4. Is the manuscript presented in an intelligible fashion and written in standard English?

Reviewer #1: No

Reviewer #2: Yes

**********

Reviewer #1: Dear authors,

I consider your study topic in general interesting and timely, but I find that there are too many short comings that needs to be addressed. Some methodological problems with your experiments that you need to carefully consider. This is not invalidating your results but it needs to be addressed openly throughout the manuscript since it affects interpretation.

Actually the biggest problem with your manuscript in my opinion is that you study faunal influence on biogeochemistry, without consideration of biogeochemical processes. This doesn't work.

Please find detailed comments in the attached document

Reviewer #2: This manuscript reports benthic fluxes of oxygen and nutrients along a gradient in ice cover, and considers the role of benthic community structure in governing these fluxes. Relatively few such measurements exist for Arctic waters, and the location of the study is interesting given the proximity to the ice edge, the depth, and the hydrography. Overall, I think the data are valuable and the methodology of the study and analyses is sound. My major suggestions are focused mainly on 1) reorganizing the manuscript such that the results and discussion link better to the stated objectives to more clearly address them, 2) lack of justification for some of the claims made in the discussion. I do think the manuscript would benefit from a thorough editorial revision to remove unnecessary wordiness and improve flow. As an example, phrases like “other studies have shown that….” are over-used and could be deleted to make the manuscript more readable. Paragraph breaks in the Discussion also occur in weird places, and several paragraphs lack topic sentences so that the point being made is unclear.

Results

- The authors report that the proportion of deposit-feeders is an important driver of fluxes; however, the data on the community structure at these sites is only contained in supplemental tables, with little granularity as weighted means. It would be helpful to show at least the proportion of the different functional groups among the different stations in the main text as a figure so the reader could get a sense of how communities vary among the sites, which in itself provides a lot of info on what the environment is like.

There is no abundance or biomass information presented in the results to give a sense of how distribution of biomass among taxa affected the results, even though CWMs turned out to be significant in the analysis. For example, are deposit feeders important because they are deposit feeders, or is it just that they were the most abundant (or largest) organisms and therefore had an outsized effect on fluxes? How did average body size vary (or not) among sites? This consideration of the differences in communities across the sites should be better incorporated into the discussion of why fluxes vary among sites, especially given that the stated aim of the paper is to consider functional diversity as a control on fluxes. There is literature on how specific species or types of deposit feeders affect nutrient fluxes as well (including direction of flux), which is not considered here.

There are two functional types of deposit feeders listed in table 2, but only “deposit feeders” appears in the analysis. Are they lumped together for the stats?

How does the size of deposit feeders matter impact the results? Because “CWM of small organisms” is also identified as important but there is no explanation of what that means exactly.

- The Results section is organized around the statistical methods used, and not around the questions asked. I suggest reorganizing based on the stated objectives at the beginning. For example, the two sections on “spatial variation” could be combined, and the “variance partitioning” section doesn’t need to stand alone. A suggested organization might be 1) Variation in flux among sites, and 2) Drivers of flux (be that environmental or faunal driven). It would make the presentation of results more impactful and clearer.

Discussion

- I personally like to see a brief summary statement at the start of the discussion that highlights key results, which makes for easy reference and indicates what the authors thought were the most important take-away messages as the results section can somethings obscure the key messages with all the technical details.

- The first section of the results (beginning line 341) goes into some detail comparing the values measured here to values measured elsewhere. While this is important information to include, what is the actual point being made in this paragraph with respect to the objectives of the project? There is no topic sentence for this section and it reads like an annotated bibliography.

- Minor points:

- Don’t begin a whole paragraph with “however”, which implies you just said something else (e.g. line 347). “However” links ideas together, and thus there should be no paragraph break before it.

- The last line of the first paragraph, beginning line 357, is circular—“the very low quantity….was very low”. Should be rephrased

- Line 361: here again there is no topic sentence. What is the point being made in this paragraph? State that point, then back it up with the details/references.

- Line 369: “sediment traps are increased in this region”—do you mean “sediment trapping?” I don’t understand this line. “particle accumulation and advection optimize conditions for sinking fluxes”—aren’t accumulation and advection the opposite of each other?

- Line 375: Again, starting with “similarly”, as with “however,” links to another idea, yet this is the beginning of a paragraph. It hasn’t been clearly stated that benthic fluxes are “heterogeneous” in this region, if that is the point being addressed, yet it is implied that this point has been made by discussing similarity to something else.

- Line 378: “explain the patterns found” What patterns? First, describe the patterns you are referring to.

I agree that you need to consider the potential role of faunal components not measured, but this passing reference to micro and meiofauna doesn’t really do that point justice. This could be expanded upon a bit to better consider their role.

- A new section on environmental drivers is not needed. In the “Variability” section, you should more explicitly describe the variability you refer to, and then follow up with an explanation of the possible reasons for that variability (environmental or biological drivers)—these aren’t separate ideas that merit separate subheaders.

- Section beginning line 394. Seems to suggest sea ice is important because it mediates grain size? But doesn’t it also mediate organic matter flux, which would seem quite important to remineralization rate AND community structure?

- I also wonder if the effect of sea ice in the analysis is driven entirely by the one station nearest the ice, which seems to sit apart from everything else in the ordination plots.

- Last few paragraphs beginning line 442: In my opinion, this is the most interesting part of the study, but there are factually incorrect statements and tenuous assertions made. In some cases, the references are not appropriate for some of the statements made.

For example:

- line 444: the role of deposit feeders in remineralization is less understood—really? There is ample literature from all over the globe on this topic

- Line 445: “deposit feeders acquire food by swallowing large volumes of sediment”. This is not correct, and the reference given refers to a paper on feeding on ice algae. There is ample literature on the many and varied strategies of deposit feeders and their ecology. Moreover, the reader must go to the appendices to try and tease out what strategies are common in this study location because the taxonomic identities of the sampled fauna are only given in a supplemental table, and the distribution of functional modes among stations is not described or depicted in a figure in the main text.

- Lines beginning at 447- yes it is true that there is evidence of feeding on ice algae elsewhere. However, it is not really clear whether this is due to preference for ice algae (as the authors claim on line 460), or whether deposit feeders just eat whatever is available and abundant where they are. I think it is a tenuous argument to assert (as on line 456) that deposit feeders somehow have an advantage over other feeding modes in the presence of ice algae, or that this supposed preference (line 460) is the reason why deposit feeders have “chosen” to live in this area. It is much more likely that the other environmental characteristics (e.g. grain size) favor the presence of deposit feeders as is the case with most deeper soft-sediment areas.

- Line 466: “Together, these results…” – Which results is this referring to?

- There is no consideration in this section of which types of deposit feeders were found in this study at these sites, yet there is reference to specific modes (e.g. lines 467 – 469; 473 - 475) found to be important in other areas. What is the link to the data from this study? Are these modes found in this dataset? Explore this idea further, and again bring in the specific results about the community to the results section.

- Line 480 – 483: This is a run-on sentence and confusing to read.

- Line 490 refers to “two distinct regions” that were identified. I missed where this was presented in the results?

- Line 497: Our findings provide crucial support… Explain further, identifying clear links between these data and “assessing the ability of benthos to process carbon in response to climate change”. These results were not presented in the context of climate change per se.

- Last line of conclusions is unnecessary and should be deleted.

**********

what does this mean?). If published, this will include your full peer review and any attached files.

If you choose “no”, your identity will remain anonymous but your review may still be made public.

**Do you want your identity to be public for this peer review?**  For information about this choice, including consent withdrawal, please see our Privacy Policy

Reviewer #1: No

Reviewer #2: No

**********

[NOTE: If reviewer comments were submitted as an attachment file, they will be attached to this email and accessible via the submission site. Please log into your account, locate the manuscript record, and check for the action link "View Attachments". If this link does not appear, there are no attachment files.]

To ensure your figures meet our technical requirements, please review our figure guidelines: https://journals.plos.org/plosone/s/figures

You may also use PLOS’s free figure tool, NAAS, to help you prepare publication quality figures: https://journals.plos.org/plosone/s/figures#loc-tools-for-figure-preparation.

NAAS will assess whether your figures meet our technical requirements by comparing each figure against our figure specifications.

Attachment Submitted filename: Review Combaz et al.docx

# Author comment: Author response to Decision Letter 1

**DOI:** 10.1371/journal.pone.0345559.r002

3 Sep 2026

We sincerely thank the Academic Editor and the reviewers for their careful and constructive comments. We have considered all comments and implemented the necessary revisions throughout the manuscript. In addition to addressing each specific point raised in the reviews, we have substantially revised the manuscript to improve its overall coherence and alignment with the objectives of the study. In particular, we have strengthened the biogeochemical context, clarified and expanded the Methods to improve transparency and ensure better alignment with the research objectives, reorganized and streamlined the Results, and comprehensively revised the Discussion to improve its structure, flow, and interpretation.

We have also added a new figure presenting benthic community composition across stations, generated a new Figure 1 map using only publicly available data, and updated the variation-partitioning figure to include the environmental component. All changes made in response to the comments are detailed point-by-point in the accompanying "Response to Reviewers" document and are reflected in the revised manuscript files. We believe that these revisions have substantially improved the clarity, rigor, and overall presentation of the manuscript.

Attachment Submitted filename: Response to Reviewers.docx

# Editor report: Decision Letter 1

**DOI:** 10.1371/journal.pone.0345559.r003

**Authors:** Clara Rodrigues

22 Oct 2026

Hydrographic and sea ice gradients shape benthic ecosystem functioning through sediment characteristics and benthic functional structure in Nares Strait, Canadian Arctic

PONE-D-26-11399R1

Dear Dr. Combaz,

We’re pleased to inform you that your manuscript has been judged scientifically suitable for publication and will be formally accepted for publication once it meets all outstanding technical requirements.

Within one week, you’ll receive an e-mail detailing the required amendments. When these have been addressed, you’ll receive a formal acceptance letter and your manuscript will be scheduled for publication.

An invoice will be generated when your article is formally accepted. Please note, if your institution has a publishing partnership with PLOS and your article meets the relevant criteria, all or part of your publication costs will be covered. Please make sure your user information is up-to-date by logging into Editorial Manager at Editorial Manager® and clicking the ‘Update My Information' link at the top of the page. For questions related to billing, please contact billing support.

If your institution or institutions have a press office, please notify them about your upcoming paper to help maximize its impact. If they’ll be preparing press materials, please inform our press team as soon as possible -- no later than 48 hours after receiving the formal acceptance. Your manuscript will remain under strict press embargo until 2 pm Eastern Time on the date of publication. For more information, please contact onepress@plos.org.

Kind regards,

Clara F. Rodrigues

Academic Editor

PLOS One

Additional Editor Comments (optional):

Thank you for providing a comprehensive and well-structured response.

Reviewers' comments:

# Editor report: Acceptance letter

**DOI:** 10.1371/journal.pone.0345559.r004

**Authors:** Clara Rodrigues

PONE-D-26-11399R1

PLOS One

Dear Dr. Combaz,

I'm pleased to inform you that your manuscript has been deemed suitable for publication in PLOS One. Congratulations! Your manuscript is now being handed over to our production team.

At this stage, our production department will prepare your paper for publication. This includes ensuring the following:

* All references, tables, and figures are properly cited

* All relevant supporting information is included in the manuscript submission,

* There are no issues that prevent the paper from being properly typeset

You will receive further instructions from the production team, including instructions on how to review your proof when it is ready. Please keep in mind that we are working through a large volume of accepted articles, so please give us a few days to review your paper and let you know the next and final steps.

Lastly, if your institution or institutions have a press office, please let them know about your upcoming paper now to help maximize its impact. If they'll be preparing press materials, please inform our press team within the next 48 hours. Your manuscript will remain under strict press embargo until 2 pm Eastern Time on the date of publication. For more information, please contact onepress@plos.org.

You will receive an invoice from PLOS for your publication fee after your manuscript has reached the completed accept phase. If you receive an email requesting payment before acceptance or for any other service, this may be a phishing scheme. Learn how to identify phishing emails and protect your accounts at https://explore.plos.org/phishing.

If we can help with anything else, please email us at customercare@plos.org.

Thank you for submitting your work to PLOS One and supporting open access.

Kind regards,

PLOS One Editorial Office Staff

on behalf of

Dr. Clara F. Rodrigues

Academic Editor

PLOS One
